# Panax notoginseng: derived exosome-like nanoparticles attenuate ischemia reperfusion injury via altering microglia polarization

**DOI:** 10.1186/s12951-023-02161-1

**Published:** 2023-11-10

**Authors:** Shiyi Li, Ru Zhang, Anni Wang, Yang Li, Miaomiao Zhang, Jisu Kim, Ying Zhu, Qizheng Wang, Yue Zhang, Ying Wei, Jianxin Wang

**Affiliations:** 1 Department of Pharmaceutics, School of Pharmacy, Fudan University & Key Laboratory of Smart Drug Delivery, Ministry of Education, Shanghai, 201203 China; 2 Department of Integrative Oncology, Fudan University Shanghai Cancer Center, Shanghai, 200032 China; 3 Department of Integrative Medicine, Huashan Hospital, Fudan University, Shanghai, 200040 China; 4 Institutes of Integrative Medicine, Fudan University, Shanghai, 201203 China

**Keywords:** Panax notoginseng, Exosome-like nanoparticles, Ischemic stroke, Cerebral ischemia reperfusion injury, Microglia polarization

## Abstract

Cerebral ischemia/reperfusion (CI/R) injury is a clinical conundrum during the treatment of ischemic stroke. Cell-derived exosomes (CDE) were proved to be therapeutically effective for CI/R injury. However, production of CDE is time and effort consuming. Increasing studies reported that plants can also generate exosome-like nanoparticles (ELN) which are therapeutically effective and have higher yield compared with CDE. In this study, a commonly used Chinese herb *Panax notoginseng* (PN), whose active ingredients were well-documented in the treatment of CI/R injury, was chosen as a source of ELNs. It was found that Panax notoginseng derived exosome like nanoparticles (PDN) could enter the brain without modification and ameliorate cerebral infarct volume, improve behavior outcome and maintained the integrity of BBB. PDNs attenuated CI/R injury by altering the phenotype of microglia from “pro-inflammation” M1 type to “anti-inflammation” M2 type. Also, we found that lipids from PDNs were the major therapeutic effective component. As a mechanism of action, PDN was proved to exert therapeutic effect via activating pI3k/Akt pathway.

**Graphical Abstract:**

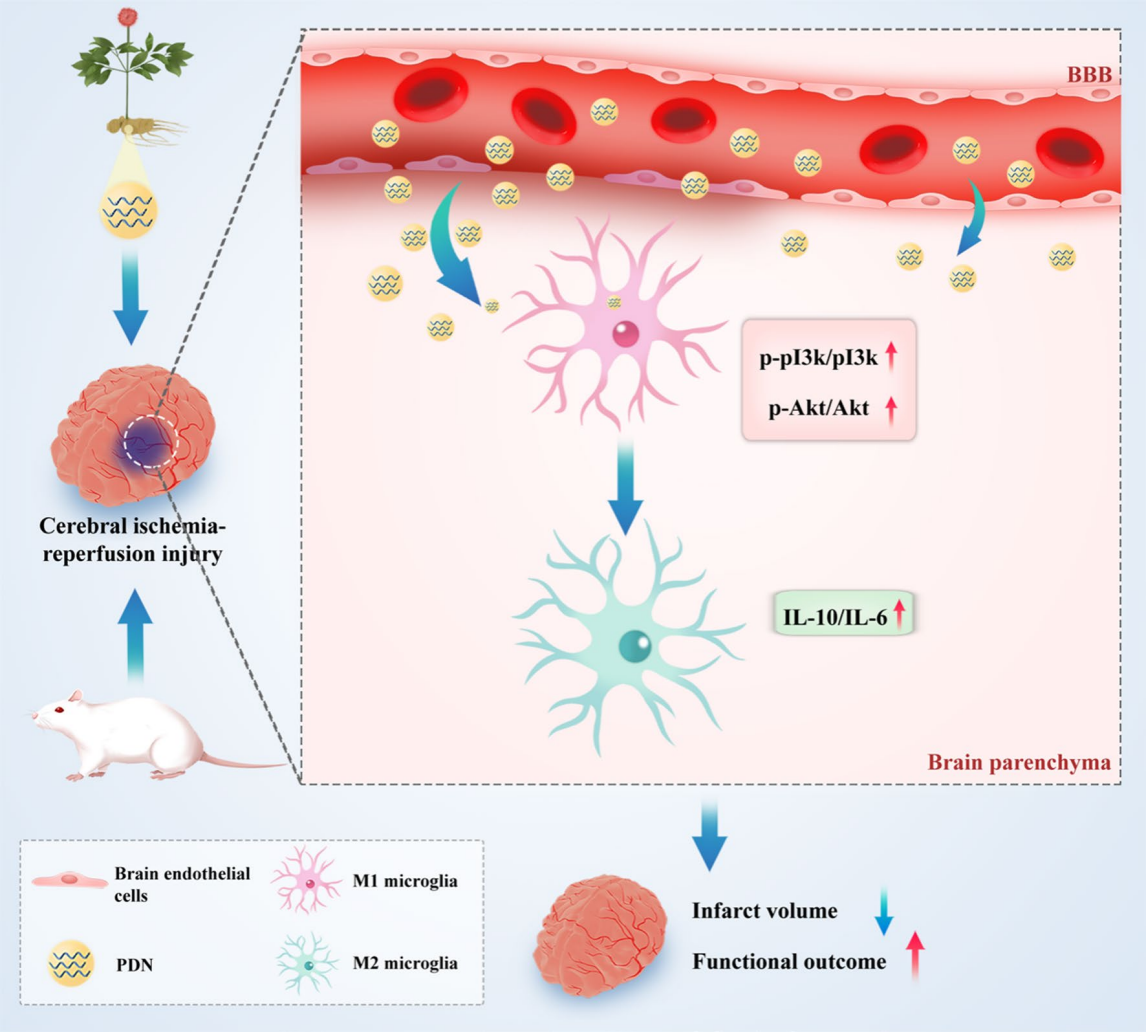

**Supplementary Information:**

The online version contains supplementary material available at 10.1186/s12951-023-02161-1.

## Introduction

Cerebral ischemia (CI) is a cardiovascular disease with a high rate of morbidity and mortality. Up to now, the most common clinical regimen for CI is thrombolysis treatment, which restore blood and oxygen supply for the damaged brain. However, sudden recanalization of the occluded artery causes production of reactive oxygen species (ROS), initiates inflammation and leads to secondary cytotoxicity of the already damaged tissue, which is called cerebral ischemia/reperfusion (CI/R) injury [1, 2]. Inflammation takes place at the acute stage of stroke (within minutes to hours) and is probably the predominant mechanism of injury within hours, which can induce downstream reaction lasting for several days [3]. Clinically, several anti-inflammation drugs which target neutrophil recruitment have been developed as potential therapies for IS. For example, a mAb to ICAM-1 [4], a humanized antibody to the CD11b/CD18 [5] and the UK-279267 [6] have been tested in clinic. However, all these trials were unsuccessful as a result of lack of neuroprotective efficacy and side-effects such as leukopenia and immunosuppression [7]. Hence, development of effective and secure anti-inflammation therapies is of urgent need.

Microglia are residential immune cells in central nervous system and account for maintaining the homeostasis of brain microenvironment [8], and thus have been proved to be the major player of inflammation during CI/R progress. Under pathological condition, microglia change their morphology and function according to the surrounding microenvironment and disease progress. Researchers classified microglia into two subtypes based on their function, i.e., M1 pro-inflammatory subtype and M2 anti-inflammatory subtype [9]. When CI/R takes place, most microglia are initially M2 type which help remove cell debris. With the disease develops, microglia polarize into M1 subtype and exacerbate inflammation in brain tissue, which intensifies the damage and prolongs the injury period [9–11]. Mounting evidences have confirmed that altering M1 microglia into M2 subtype to inhibit microglia-induced inflammation is an effective strategy to alleviate CI/R injury [12–15].

Regimens aimed to reduce CI/R-induced inflammation have been reported and reached success in animal models [16]. However, none of them has been applied in clinical practice up to now [17, 18]. The main reason was that post CI/R, the highly restrictive nature of blood brain barrier (BBB) and the “no reflow” phenomenon made it hard for the drugs to enter the brain parenchyma. To address this, drug delivery systems using nanotechnology have been developed and showed promising effect [19, 20]. However, delivering enough therapeutic agents into the brain still remains a huge challenge for nanomaterials. Kozlovskaya et al. [21] demonstrated that most nano-systems could only deliver less than 1% (median value) of injected dose into the brain. Also, to achieve brain penetrating effect and prolonged retention, drug-delivery systems usually need delicate modification with peptide or antibody, which is too costly and laborious to apply in clinic.

Meanwhile, interest in exosomes as carriers of functional proteins, lipids and nucleic acids has been growing rapidly. Exosomes are natural nano-sized vesicles which communicate among cells by carrying around functional materials [22–24]. Exosomes derived from mesenchymal stem cell and M2 microglia have already been proved to be beneficial for CI/R recovery [25, 26]. Characteristically, exosomes have natural ability to cross the BBB without modification [27, 28] and protect its cargo protein and nucleic acid from degradation. However, cell-derived exosomes have a low yield, and it often takes 3–4 days to obtain them under laboratory condition. To address this, researchers turned to fresh plant tissues for an alternative and found that nanoparticles can also be isolated from fresh plant tissue [29]. These nanoparticles resemble exosomes both in morphology and composition. More intriguingly, they can be produced in larger scale and in only one day [30]. Plant-derived exosome-like nanoparticles (PDELNs) isolated from ginger, broccoli and ginseng were proved to be effective in the treatment of inflammatory bowel disease [30] and cancers [31, 32]. PDELNs were stable in gastrointestinal tract and protected its cargo from degradation [30], rich in therapeutic effective molecules [33] and could cross biological barrier such as intestinal tract barrier [29]. However, the transport of PDELN through the BBB and their effect on CI/R injury has not been reported yet.

*Panax notoginseng* (PN) is one of the most commonly used Chinese traditional medicine and has a long history in treating CI/R. A meta-analysis on randomized controlled trials demonstrated that *Panax notoginseng* saponins (PNS) was effective in treating acute ischemic stroke on ameliorating neurological deficit, improving activities of daily living function, and enhancing antiplatelet effects [34]. PNS is a mixture of multiple therapeutic effective saponins and each of them exerts distinct effect in the treatment. For example. ginsenoside Rd was reported to improve CI patient’s disability on 90-day post stroke in a clinical trial, by suppressing microglial proteasome-mediated inflammation [35]. Another PNS component Rg1 were proved to protect against CI/R injury through anti-oxidant activity and associated apoptosis, regulate energy metabolism and induce neurological cell proliferation [36]. However, the chemical components can only reflect part of pharmacological activities of PN and have limited effect on CI. What’s more, potential therapeutic effective materials such as amino acids, proteins and nucleic acids were discarded during the preparation of PNS. Hence, their effect is yet to be elucidated. Based on the multiple therapeutic benefits of PN on CI/R, we suppose that PN-derived exosome-like nanoparticles (PDNs), which can maintain the biological functions of PN, will be a potential candidate for CI/R treatment.

In this study, we isolated ELNs from fresh root of PN and characterized PDNs. It was found that PDNs can cross the BBB and were efficiently internalized by microglia. Moreover, PDN decreased infarct volume and improved behavior outcome in a transient middle cerebral middle artery occlusion (tMCAO) model rat. PDN alleviated CI/R injury via altering the microglia phenotype, and lipids from PDNs were the major active component of the anti-inflammatory effect. Overall, we explored PDN as a promising candidate for treating CI/R injury. And to our knowledge, this is very first study exploring PDELNs’ effect on brain disease.

## Materials and methods

### Animals and materials

Male Sprague–Dawley rats (250–270 g) were purchased from Shanghai Sipper BK Laboratory Animals Co. Ltd. (Shanghai, China). All rats were raised in the animal holding unit in Fudan University and were provided with free access to food and water under controlled conditions (12/12 h light/dark cycle with humidity of 60% ± 5%, and a temperature of 22 ± 3 °C). All animals were treated according to the Guide for the Care and Laboratory Animals and all experiments were approved and performed according to the guidelines of the Ethics Committee of Fudan University. All rats were anesthetized by intraperitoneally injected with isoflurane. 1,1ʹ-dioctadecyl-3,3,3ʹ,3ʹ-tetramethylindodicarbocyanine perchlorate (DiD), 1,1ʹ-dioctadecyl-3,3,3ʹ,3ʹ-tetramethylindotricarbocyanine iodide (DiR), ginsenoside Rg1 (purity > 90%), panax notoginsenoside Rb1 and R1(purity > 90%) were purchased from Dalian Meilun biotechnology Co. LTD. Antibodies for NeuN, vWF, Iba-1 and GAFP were purchased from Servicebio Co. Ltd. Fluorescence coupled antibody for CD86 and CD206 were purchased from BioLegend Co. Ltd.

### Isolation and characterization of PDNs

Fresh root of PN was purchased from Wenshan, Yunnan province of China. The root was washed with running water to remove mud and grounded thoroughly with phosphate buffer solution (PBS) with a ratio of 3:1 (v/w). After grinding, the mixture was filtered with gauze to remove large debris. The resulting solution was centrifuged at 2,000g for 20 min, and the supernatant was then centrifuged again at 10,000g for 1h to get dark brown and transparent supernatant solution. Next, 2 mL of 68% (w/v) glucose solution was added to the tube and 2 mL of 27% (w/v) sucrose solution was carefully layered on it to form a cushion. The supernatant from last step was layered on the cushion and centrifuged at 100,000g for 1.5 h. After centrifugation, the band over 68% sucrose cushion was collected and referred to as crude PDNs. To purify the PDNs, the band was laid on an 8%/30%/45%/60% (2 ml each) sucrose cushion and centrifuged at 200,000g for 1.5 h. Finally, the band between 30%/45% sucrose was collected and referred to as PDNs. All the centrifugations mentioned above were carried out under 4 °C. The PDN solution was freeze-dried and stored at – 80℃ until further use.

The size and zeta potential of PDNs were measured by dynamic light scattering detector (Zetasizer, Nano-ZS, Malvern, UK). The morphology of PDNs was visualized using transmission electron microscopy (TEM, Tecnai G2 F20 S-Twin, FEI, USA) as previously described [37]. Briefly, PDNs were immobilized on a formvar-coated copper grid and negatively stained with uranyl acetate (1%, w/v). The protein concentration was quantified using a BCA protein assay kit (Beyotime Biotechnology, China) according to manufacturer’s instructions. In this paper, the dose of PDN was all presented as the weight or concentration of protein when applied to animals or cells.

The proteins and lipids of PDNs were extracted from PDN samples with RIPA protein isolation kit by Bligh and Dyer method [38], respectively. Later, the composition by LC–MS/MS (Q Exactive mass spectrometer, Thermo Scientific) coupled to Easy nLC (Proxeon Biosystems, now Thermo Fisher Scientific). The raw data of MS for each sample were combined and searched using the MaxQuant 15.5.3.17 software for identification and quantitation analysis.

### Labeling of PDNs

PDNs were labeled with 1,1'-dioctadecyl-3,3,3ʹ,3ʹ-tetramethylindodicarbocyanine perchlorate (DiD) (meilun biological co., LTD., Dalian, China) or 1,1ʹ-dioctadecyl-3,3,3ʹ,3ʹ-tetramethylindotricarbocyanine iodide (DiR) (Meilun biological Co. LTD., Dalian, China). DiD (5 μg/mL) or DiR (5 μg/mL) was added to the PDNs suspension and incubated for 30 min at 4 °C and the dissociated dye was removed using G50 Sephadex Column (GE Healthcare, Sweden).

### Biodistribution assays

To analyze the in vivo biodistribution of PNDs, DiR-labeled PDNs were intravenously administrated into rats. Near-infrared fluorescence images were obtained 1, 2, 4, 8, 12 and 24 h post injection. For ex vivo analysis, the rats were sacrificed and major organs were collected 24 h post administration. The intensity of DiR labeled PDNs in vivo and in ex vivo organs were measured using an in vivo imaging system (IVIS Spectrum, Caliper, USA).

### Therapeutic effect of PDNs in tMCAO model rats

An experimental CI/R model was established as previously described [39]. Briefly, rats were anesthetized with 10% chloride hydrate (40 mg/kg) through intraperitoneal injection. The common carotid artery (CCA), external carotid artery (ECA) and internal carotid artery (ICA) were exposed through an incision in the middle of the neck. A filament with a rounded tip aƒnd a nylon suture (Jialing, Guangzhou) was introduced into the ECA through the CCA to block the origin of ICA. 90 min later, the nylon suture was withdrawn to allow reperfusion. Two hours post reperfusion, rats from different groups were treated with PBS, Xuesaitong (XST) and 3 mg/kg PDNs, respectively.

Seventy-two hours post reperfusion, the animals were anesthetized with 10% chloride hydrate and perfused with PBS through heart. The brains were taken out and frozen under -20℃ for 15 min. After freezing, the brain was sliced into coronal sections (2 mm) using a blade and immersed in a saline solution containing 2% 2,3,5-triphenyltetrazolium chloride (TTC; Sigma, St. Louis, MO, USA) for 10 min at 37 °C, then fixed with 4% paraformaldehyde solution. Stained brain slices were photographed using a digital camera (Olympus, Germany) and quantified using Image J (Image J 1,38X; NIH, Bethesda, MD, USA). The infarct area in each slice was presented as the percentage of infarct area to the area of the whole brain slices.

Neurobehavior tests were conducted 3 days after tMCAO by investigators blinded to the experimental design. Neurological outcome was evaluated according to the modified neurological severity score (mNSS) criterion, which is an integrated score of motor function, reflex and balance tests [40]. The severity was graded in a range of 0 to 14 and a higher score stands for more severe neurological injury [41].

The apoptosis rate of brain cells was detected using the TdT-mediated nick-end labeling (TUNEL) assay kit (Servicebio, Wuhan, China) according to the manufacturer’s protocol.

FACS was used to evaluate the phenotype of microglia in the brain. Briefly, 3 days post reperfusion, rats’ brains were dissected. To prepare single cell suspension of rat brain, brain tissues were cut into 1mm^3^ and then digested with 8 U/mL papain for 15 min at 37 °C, and then meshed through 70 μm cell strainer. Cells were blocked with 3% bovine serum albumin for 30 min at 4 °C and then stained with Alexa Flour 700 anti-rat CD45 (Biolegend), FITC anti-rat CD11b/c antibody (Biolegend), PE anti-rat CD86 (Biolegend) and APC anti-rat CD206 antibody(Biolegend). After washing with PBS thrice, cells were subjected to FACS. The CD11b^hi^, CD45^low^ subgroup was identified as microglia. The catalogue numbers of antibodies were as follow: Alexa Fluor@700 anti-rat CD45 (BioLegend, Cat:202218), FITC anti-rat CD11b/c (BioLegend, 201805), PE anti-rat CD86 (BD Bioscience, Cat:551396) and af647 anti-rat 206 (Santa Cruz, Cat:sc-58986).

### *Primary microglia isolation and *in vitro* oxygen/glucose deprivation model*

Primary microglia were isolated from neonatal SD rats (< 24h). Briefly, cortex was dissected and trypsinized with 0.025% Trypsin under 37 °C for 15 min. Then the cells were plated on poly-l-lysine coated T75 flask. Five days later, primary microglia were isolated by shaking the flask at 200 rpm for 4h and plated in new well plates.

To optimize PDNs’ concentration for treating microglia, primary microglia were seeded into a 96 well plate at a density of 5000 cell per well, and a series of 20, 10, 5, 2.5, 1.25 μg/ml PDN was given to primary microglia and incubated for 24 h. Afterwards, cck8 solution (Meilunbio, Dalian, China) was added into the well and incubated for another 1 h, and absorbance 450 nm was measured by a microplate reader.

In vitro oxygen/glucose deprivation reperfusion (OGD/R) model were established as follow. Briefly, culture medium was changed into glucose-free DMEM without FBS. Then, the cells were placed in a chamber with a continuous flux of gas (94% N_2_/5% CO_2_/1% O_2_) for 2 h to mimic the hypoxic status. Then, for “reperfusion”, cells were transferred into normal chamber and medium was changed into high-glucose DMEM supplemented with 10% FBS. Cells were pretreated with PDN 2 h before OGD and during the whole process of OGD/R. Twenty-four hours post reperfusion, the cells were collected for further measurement of gene expression and fluorescence-activated cell sorting (FACS) analysis.

For FACS analysis, microglia were digested by trypsin and resuspended in cold PBS. The cells were stained with monoclonal antibodies, anti-CD206 and anti-CD86, to detect microglia surface markers. For each sample, at least 1 × 10^4^ cells were analyzed by flow cytometry. Data was analyzed by FlowJo software (BD Biosciences, USA).

For RT-PCR analysis, total RNA was isolated using RNA extract kit (Servicebio, China) and ten RT-PCR was performed using FastStart Universal SYBR Green Master (Rox) (Roche, USA) following the manufacturer’s instructions and tested on an ABI Prism 7500 Sequence Detection System (Applied Biosystems, USA). Information of primer sequences are shown in Table 1. The 2^−ΔΔCt^ method was used to calculate fold changes in gene expression normalized to control.

**Table 1:** Primer sequences for real-time RT-PCR analysis

Gene	Forward	Reverse
ACTB	CAAGTGGGTGGCATAGAGG	ATGACGAAGAGCACAGATGG
IL-10	ACTGCTATGTTGCCTGCTCTTACTG	TGGGTCTGGCTGACTGGGAAG
IL-6	ACTTCCAGCCAGTTGCCTTCTTG	TGGTCTGTTGTGGGTGGTATCCTC

To investigate the bioactive components of PDNs, PDNs went through following modifications: (1) PDN-derived liposomes were made as previously described to investigate the effect of lipids in PDNs[42]; (2) PDNs were boiled at 100℃ to denature the proteins, referred to as PDN without protein (PDN w/o protein); (3) 10 μg/mL RNase were loaded into PDNs, by using ultrasonic probe (500 W, 2 s each time, 2 s gap between two sonication, 20 times, on ice), to deplete RNAs in PDNs. PDNs depleted of RNAs were referred to as PDN without RNA (PDN w/o RNA).

### Western blot analysis

For western blot analysis, treated microglia or brain tissue were lysed on ice by radio immunoprecipitation assay (RIPA) (Beyotime, China) supplemented with protease and phosphatase inhibitor cocktail (Beyotime, China) and centrifuged. Then the proteins were collected from the supernatant, and separated on 12.5% SDS-PAGE gel (Epizyme, China) according to manufacturer’s instruction. The gel was transferred to polyvinlidene fluoride (PVDF) membrane (Epizyme, China) for 1 h and target band was cut off and non-specific binding was blocked by incubating the bands with 3% bovine serum albumin (Aladdin, China) under room temperature and then incubated with primary antibody overnight. The bands were washed with TBST thrice and incubated with peroxidase-conjugated goat anti-rabbit IgG (H + L) (Yeason, China) for one hour. Later, the bands were washed thrice with TBST and incubated with Omni-ECL Pico Light Chemiluminescence Kit (Epizyme, China) according to manufacturer’s instructions. Images were taken by ProteinSimple M (FluoroChem, UK) and analyzed by Image J software (USA). Production information of primary antibodies was as follow: PI3 Kinase p110α (C73F8) Rabbit mAb #4249, hospho-PI3 Kinase p85 (Tyr458)/p55 (Tyr199) (E3U1H) Rabbit mAb #17366, Phospho-Akt (Ser473) (D9E) XP® Rabbit mAb #4060, Akt (pan) (11E7) Rabbit mAb #4685.

### Immunofluorescence evaluation of PDN distribution in the brain

Did labelled PDNs were injected into MCAO rats as described in Sect. "Therapeutic effect of PDNs in tMCAO model rats". Twenty-four hours later, brains were dissected and sliced into 2 mm sections. Later, brain sections were incubated with Iba-1(Absin, Shanghai), GFAP (Absin, Shanghai), vWF (Absin, Shanghai) or NeuN (Servicebio, Wuhan) antibody overnight at 4 ℃, then incubated with conjugated secondary antibody for 1 h at RT in the dark. After several washes with PBS, the slices were incubated with DAPI for 5 min then mounted in glycerol. Slices were imaged under a fluorescence microscope.

### Statistical analysis

Data was presented as means $\pm$ SEM. Unpaired t-tests or one-way Analysis of Variance (ANOVA) were performed for two groups or for multiple group comparisons, followed by Dunnett test. A value of P < 0.05 was considered statistically significance (*P < 0.05, **P < 0.01, ***P < 0.001).

## Results

### Isolation and characterization of PDNs

**Fig.1:**
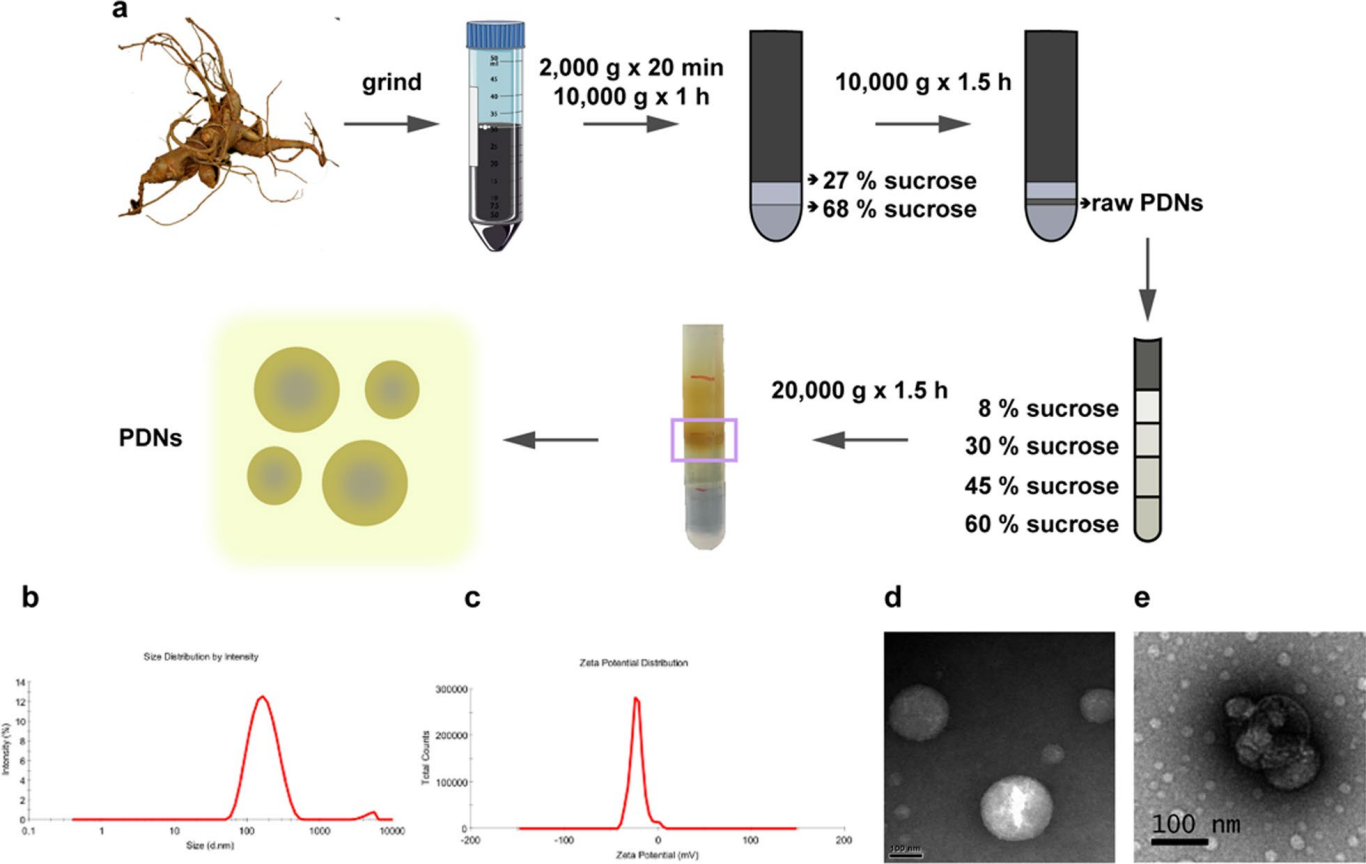
Isolation and characterization of PDNs.** a** Schematic illustration of isolating process of PDNs. **b** Size distribution of PDNs detected by DLS. **c** Zeta potential of PDNs dissolved in PBS. TEM image of PDNs **d** before and **e** after freeze-drying. Scale bar = 100 nm

PDNs were isolated and purified from homogenized root of *Panax Notoginseng* through ultracentrifugation and sucrose gradient centrifugation methods [42] with minor modification. Schematic illustration of PDNs isolating method was demonstrated in Fig. 1a that nanoparticles from the 30%/45% interface was characterized and referred to as PDNs. According to TEM and dynamic light scattering (DLS) analysis, PDNs had an average diameter of approximately 151.3 nm with a polydispersity index of 0.151 (Fig. 1b). The zeta potential of PDNs was -8 mV in PBS (Fig. 1c). TEM analysis showed that PDNs were spherical when freshly isolated (Fig. 1d) and had a cup-shape morphology after freeze-drying (Fig. 1e).

### Biochemical characterization of PDNs

It was reported that PDELNs carry proteins, nucleic acids and lipids from their origin plants. To get a full view of PDN’s chemical and biological composition, and provide further information for the follow-up studies, cargos from PDN were characterized and analyzed.

Composition analysis of PDN lipids revealed that lipids of PDN were mainly ceramide (~ 26.4% of total lipids), phosphatidic acid (~ 21.9% of total lipids), diglyceride (~ 13.1% of total lipids) and triglyceride (~ 12.8% of total lipids). In addition, most of the lipids were polyunsaturated fatty acids (PUFA, ~ 38.2%), followed by monounsaturated fatty acid (MUFA, ~ 29%), diunsaturated fatty acids (DUFA, ~ 28.7%) and saturated fatty acid (SFA, ~ 4.07%) (Additional file 1: Fig S1). Lipid class and species analysis was listed in Additional file 1: Table S1.

Proteomic analysis revealed that PDNs contain rich protein components, in total 206 different protein species (Additional file 1: Table S2). Most proteins were cytosolic (92 out of 206) and located at plasma membrane (44 out of 206). To analyze the function of proteins carried by PDNs, the proteins were subjected to analyze by Gene Ontology (GO) and classified into three categories: biological process, cellular compartment and molecular function (Additional file 1: Fig. S2).

According to MISEV2018 [43], at least three categories of proteins should be analyzed to demonstrate the existence of exosomes, which refer to (1) Transmembrane or GPI-anchored proteins localized at the external membrane of the donor cells, and plasma membrane and/or endosomes of eukaryotic cells. Their presence indicates the distinguishing lipid-layer structure of extravesicular vesicles (EV). According to the protein profiling of PDNs, 44 out of 206 proteins located at the plasma membrane, suggesting that PDNs might originate directly from the budding of plasma membrane; (2) Cytosolic proteins recovered from PDNs. 92 out of 206 detected proteins from PDNs located at cytosol. This category of proteins demonstrates that the preparation displays the structure of lipid bilayers enclosing intracellular material; (3) Some proteins that are usually co-isolated with EVs, and evaluation of this category of proteins can help to illustrate the purity of EV preparation. Based on the protein profiling, PDNs resembled exosome in protein composition, indicating its possible exosomal origin.

Deep sequencing revealed that there were 40 different miRNAs containing between 20 and 24 nucleotides (Additional file 1: Table S3). It was predicted by TargetFinder that miRNAs from PDNs potentially target and regulate the expression of total 4010 human genes by binding to their 3ʹ-untranslated regions.

### Biodistribution of PDNs in tMCAO rats after intravenous administration

**Fig. 2:**
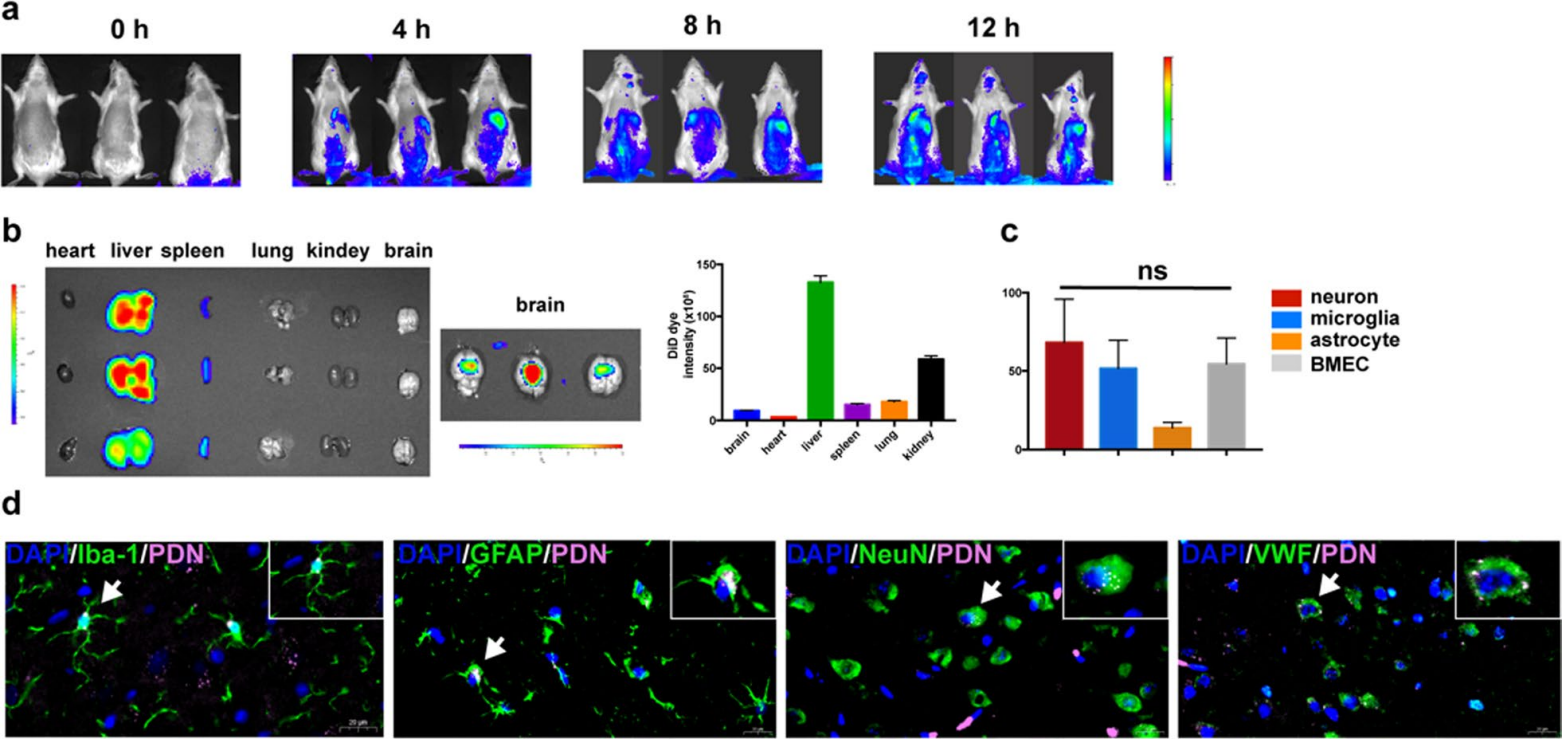
Biodistribution of PDNs in MCAO rats after intravenous administration.**a** In vivo biodistribution of PDNs 0 ~ 12 h post administration. **b** Ex vivo biodistribution of PDNs in major organs 24 h post administration. **c** Quantification of uptake efficiency of different cells in brain parenchyma. (n = 6) BMEC: brain microvascular endothelial cell. **d** Immunofluorescence images of cellular location of PDNs in the brain. Scale bar = 20 μm. (n = 3)

To determine the biodistribution of PDNs in vivo, DiR-labeled PDNs were injected into rats through tail vein and their distribution was detected by IVIS at different time points (Fig. 2a). PDNs could be observed in the brain 8 h post administration. For ex vivo imaging, the rats were killed 24 h post administration and major organs were imaged (Fig. 2b). Based on IVIS imaging result, it was demonstrated that PDNs accumulated mostly in the liver, and could be also efficiently delivered into the brain. To observe the distribution of PDN in the brain on cellular level, brain sections were prepared and stained with antibody which discriminates microglia (Iba-1), neurons (NeuN), astrocytes (GFAP) and endothelial cells (vWF) (Fig. 2d). Images were taken at 6 random views of each section. To compare the uptake efficiency of different cells, the ratio of PDN-positive cells among whole cells were calculated. It can be seen from Fig. 2c that compared with astrocytes and microvascular endothelial cells, neurons and microglia had a higher uptake efficiency of PDNs.

To determine the biocompatibility of PDN, HE staining of major organs were conducted. Based on the HE staining image, no apparent tissue or organ damage was observed in the heart, liver, spleens, lungs or kidneys of rats (Additional file 1: Fig. S3). Routine blood examination revealed that PDNs didn’t lead to changes of blood cells, hemoglobin and platelets (Additional file 1: Fig. S5). Overall, PDNs could enter brain parenchymal and efficiently internalized by neurons and microglia after i.v. administration, and had good biocompatibility in major organs.

### Effect of PDNs on CI/R injury in rats

**Fig. 3:**
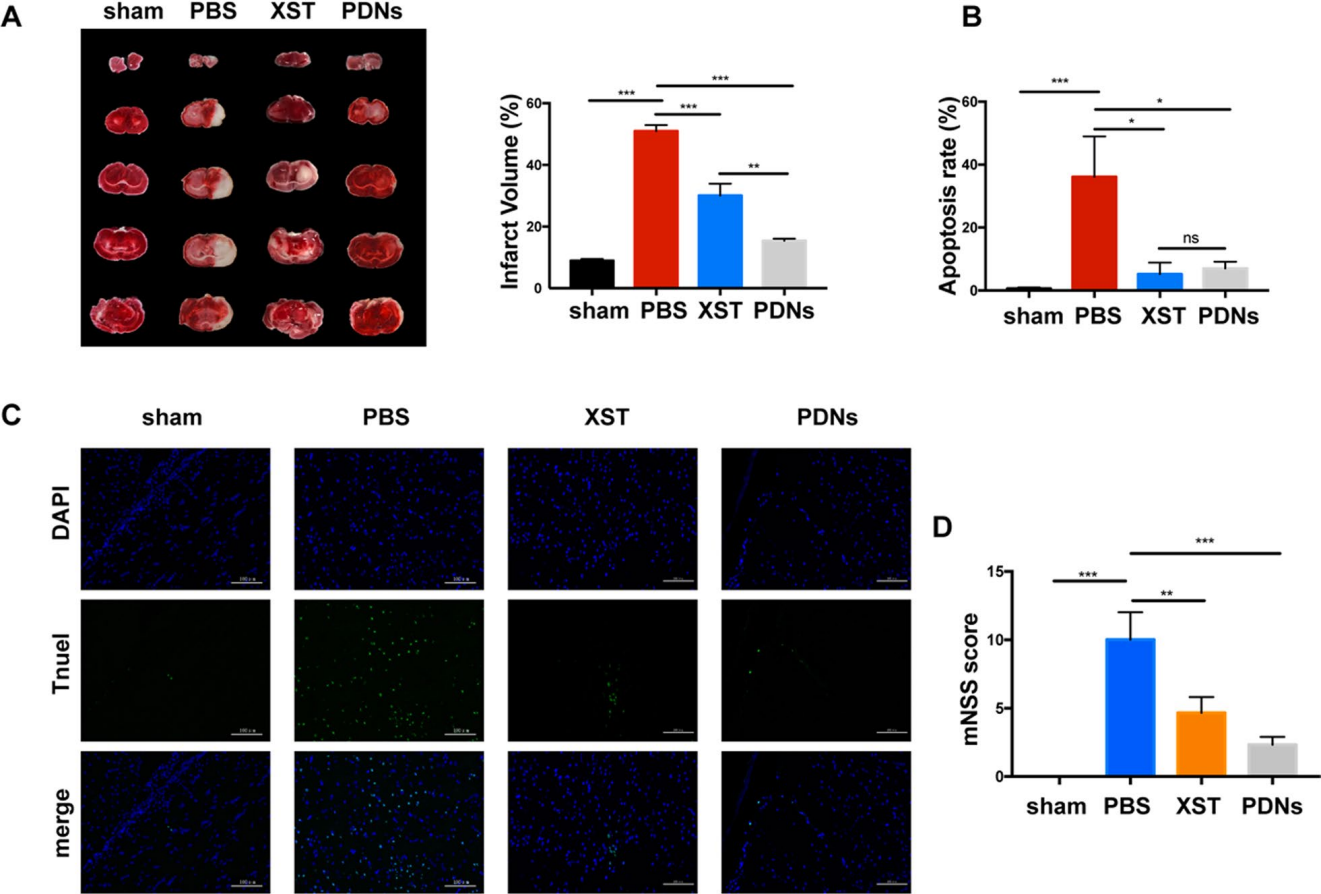
PDNs attenuated CI/R injury in rats. **a** Representative TTC staining 72 h post reperfusion and quantification of infarct volume of rats in each group. **b** Apoptosis rate of cells in peri-infarct area of ischemic brains. **c** Representative confocal image of TUNEL staining. **d** mNSS score of rats 72 h post tMCAO. Scale bar = 20 μm. Data was presented as mean ± SD (n = 3). *P < 0.05, **P < 0.01,***P < 0.001

To evaluate the therapeutic effect of PDNs on I/R injury, tMCAO model was established on SD rats and PDNs were iv. administrated 2 h post reperfusion. The dose of PDN was screened in a pilot experiment and 1.5, 3 and 6 mg/kg PDN was administrated, respectively. As shown in Additional file 1: Fig. S4, a dose of 3 mg/kg significantly decreased the infarct volume and we found 6 mg/kg PDN caused a morality of 40% in tMCAO model rats. Hence, we chose a dose of 3 mg/kg for the further investigation. Based on the TTC staining of rat brain slices, PDNs treatment significantly attenuated infarct volume by 35.5 ± 1.3% in I/R rats (Fig. 3a, b). More intriguingly, this effect was significantly higher than that of XST. Moreover, TUNEL staining revealed that both PDN and XST could suppress cell apoptosis in peri-infarct area (Fig. 3c, d).

MNSS tests was carried out to analyze the neuroprotection effect of PDNs on MCAO rats. As shown in Fig. 3d, PDNs treatment resulted in significant reduced score compared with PBS group, which demonstrated that PDNs could improve the recovery of motor and sensory functions of rats post CI/R injury.

### Effect of PDNs on inhibiting microglia-induced inflammation after CI/R

**Fig. 4:**
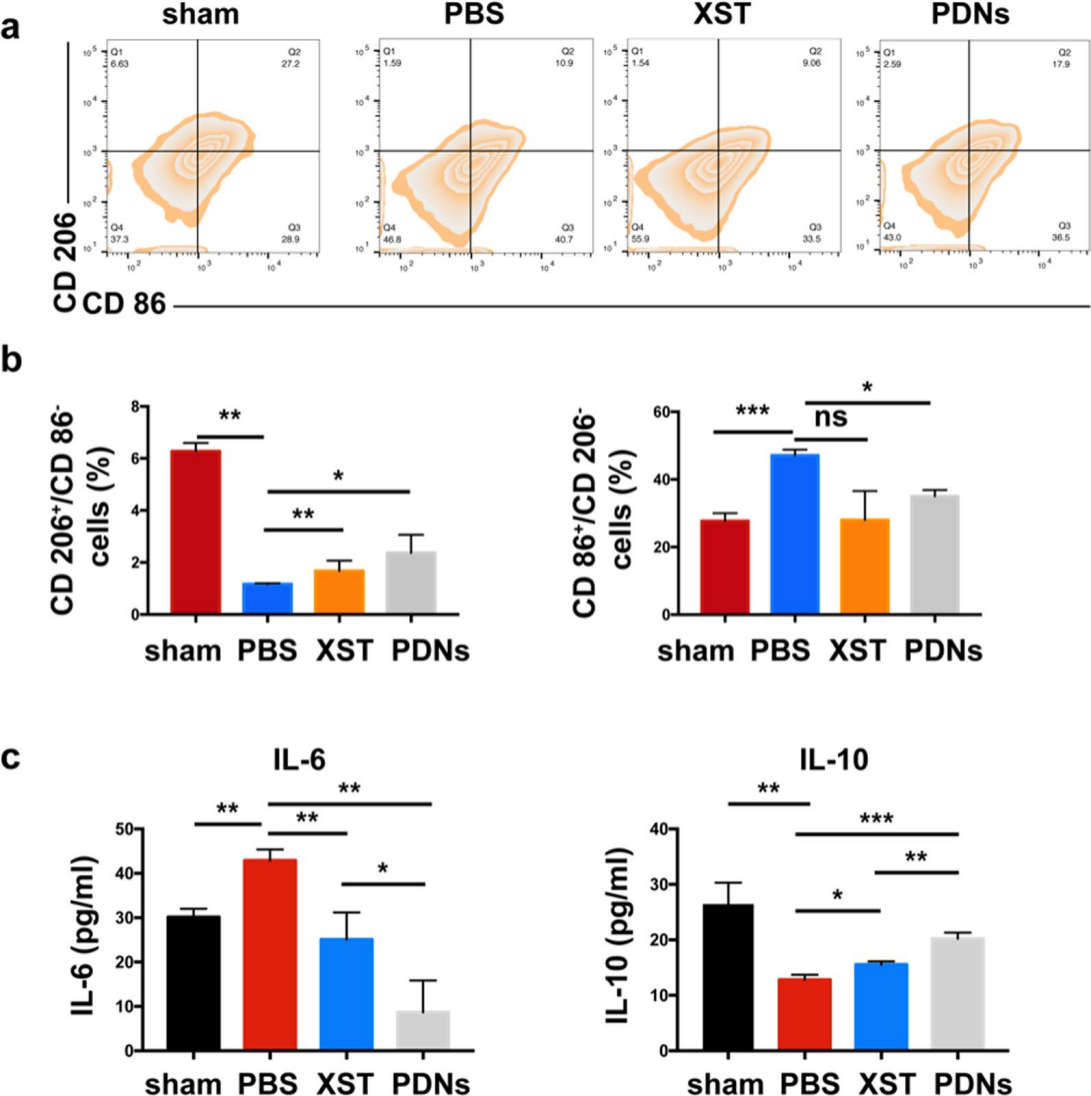
PDNs inhibited microglia-induced inflammation after CI/R. **a** Representative FACS analysis of rat brain cell suspension. **b** Quantification of proportion of M1 and M2 microglia in rat brain 72 h post tMCAO.** c** Concentration of inflammatory cytokine TNF-α, IL-6 and IL-10 in brain tissue 72 h post tMCAO. Data was presented as mean ± SD (n = 3). *P < 0.05, **P < 0.01, ***P < 0.001

Microglia is the resident immune cell in the brain under I/R condition, with different phenotypes playing opposite functions [9]. Since brain cell apoptosis, BBB dysfunction and repair, neuronal function damage and recovery are highly associated with microglia changes in CI/R, the microglia phenotype after PDN treatment were analyzed. According to the results of flow cytometry analysis, PDNs significantly reduced the percentage of CD 86^+^ CD 206^−^ microglia (M1) at 72 h post-stroke and increased CD 86^−^ CD 206^+^ microglia (M2) compared with PBS (Fig. 4a, b). To provide further evidence for microglia polarization, the concentrations of inflammatory cytokines were detected in rat brains. The level of proinflammatory cytokines TNF-α and IL-6 increased in brain tissue after CI/R while anti-inflammatory cytokine IL-10 decreased (Fig. 4c). The treatment of PDNs could reduce the concentration of TNF-α and IL-6 and increase IL-10 in brain tissue, suggesting the inhibiting effect of PDNs on microglia-mediated neuroinflammation after CI/R in rats.

### *PDNs altered primary microglia polarization after OGD/R insult *in vitro

**Fig. 5:**
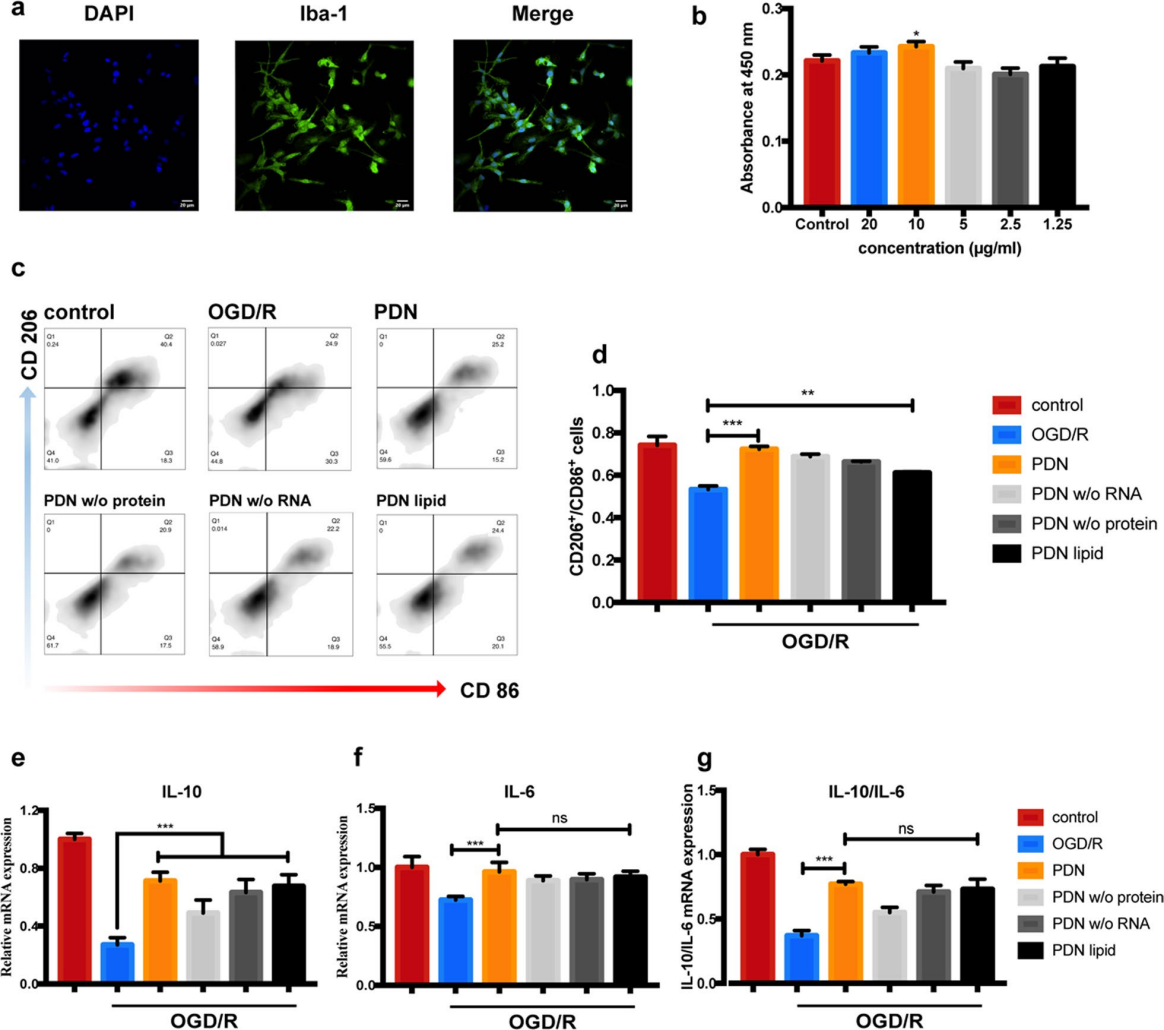
PDNs altered primary microglia polarization after OGD/R insult in vitro. **a** Representative immunofluorescence image of primary microglia. (bar = 20 μm). **b** Effect of PDNs on primary microglia’s viability. **c** Representative flow cytometry image of primary microglia after OGD/R insult. **d** Quantitative analysis of M1 and M2 microglia after OGD/R insult. Quantitative analysis of **e** IL-10 and f. IL-6 mRNA expression of primary microglia after OGD/R insult. Data was presented as mean ± SD (n = 3). *P < 0.05, **P < 0.01, ***P < 0.001

Primary microglia were isolated from neonatal SD rats and verified by Iba-1 staining (Wako, Japan). As shown in Fig. 5a, the purity of isolated primary microglia was higher than 95%.

Firstly, we optimized the PDNs’ concentration based on its effect on microglia’ viability. As a result, PDNs were not toxic to primary microglia even at a concentration of 20 μg/ml. And 10 μg/ml PDNs can slightly induce the proliferation of the cell, hence, we chose this concentration in the following experiments (Fig. 5b). In the microglia OGD model, after 2 h of OGD and 24 h of reperfusion, the ratio of CD206^+^/CD86^+^ cells significantly decreased, indicating that microglia polarized into M1 phenotype after OGD/R insult (Fig. 5c,d). Accordingly, PDN downregulated the M1-related mRNA level (IL-6) and upregulated the M2-related mRNA level (IL-10) after OGD/R insult (Fig. 5e,f).

Next, we investigated which component from PDN carried out the aforementioned effect. RNA or protein deprivation eliminated only part of PDNs’ effect. However, PDN lipid-derived nanoparticles exerted almost same effect of PDNs (Fig. 5c–f). In conclusion, PDNs’ lipid was responsible for its therapeutic effect on microglia post OGD/R insult.

### PDN altered microglia polarization through pI3k/Akt pathway

**Fig. 6:**
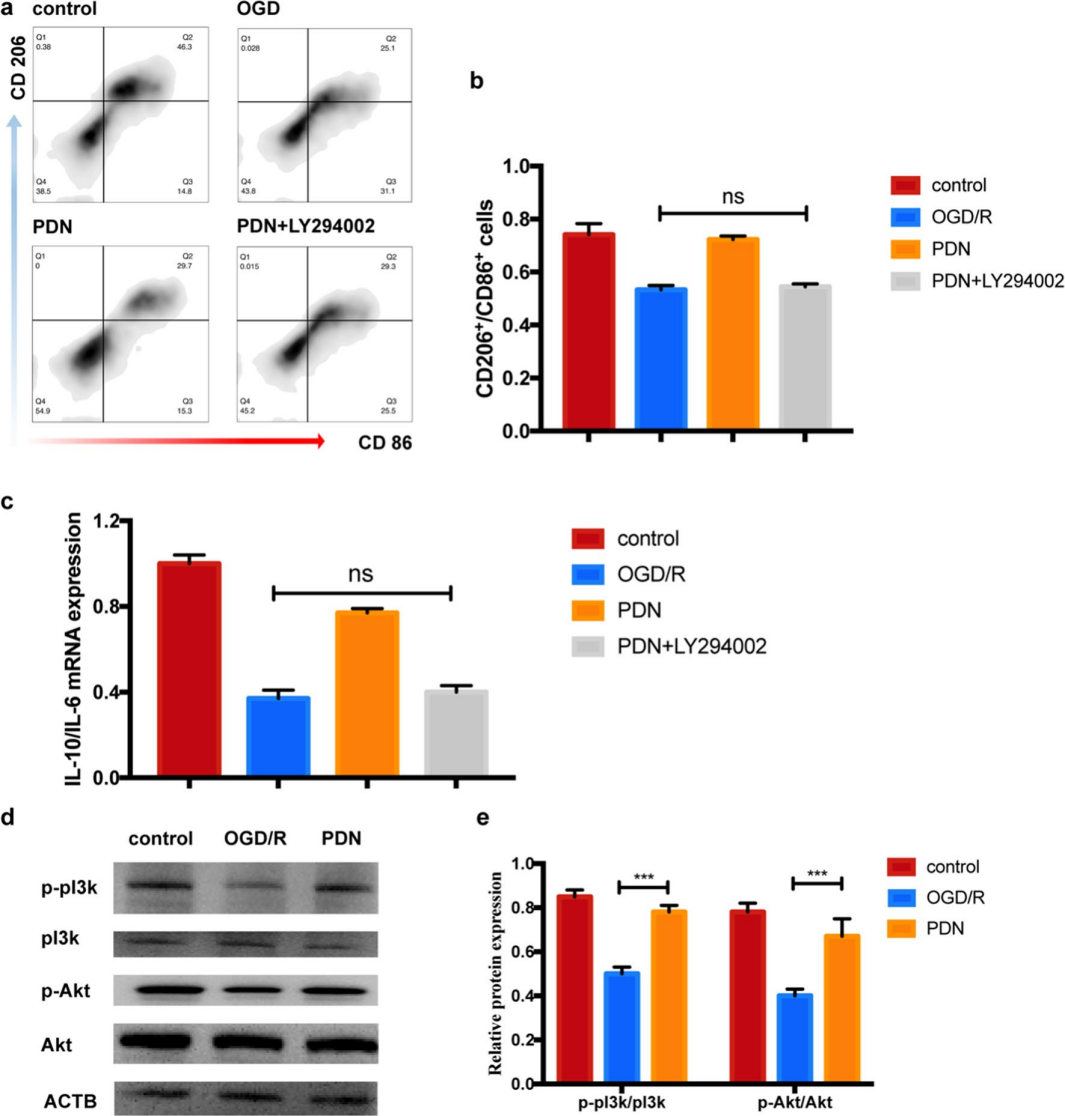
PDN altered microglia polarization through pI3k/Akt pathway. **a** Representative flow cytometry image of primary microglia after OGD/R insult. **b** Representative flow cytometry image of primary microglia after OGD/R insult. Quantitative analysis of **c** IL-10 /IL-6 mRNA expression of primary microglia after OGD/R insult. **d**, **e** Representative western blot image and quantitative analysis of pI3k/Akt pathway related protein expression. Data was presented as mean ± SD (n = 3). *P < 0.05, **P < 0.01, ***P < 0.001

Previous study demonstrated that PN saponins could downregulated the infarct volume, maintained BBB integrity and improved behavioural outcome after CI/R injury through activating the pI3k/Akt pathway [44]. Hence, we hypothesized that PDNs could alter microglia polarization through the same pathway. Resultly, LY294002 (a pI3k pathway inhibitor) pretreatment eliminated PDNs’ effect on primary microglia (Fig. 6a–c). In addition, PDN significantly upregulated the p-pI3k/pI3k and p-Akt/Akt protein ratio (Fig. 6d, e). In conclusion, PDN altered microglia polarization after OGD/R insult by activating pI3k/Akt pathway.

## Discussion

In the present study, we demonstrated that ELNs can be isolated from fresh root of PN, and PDNs can ameliorate CI/R injury and improve functional outcome in an tMCAO rat model. Furthermore, FACS analysis demonstrated that PDN alleviated CI/R injury by altering microglia polarization. It was also found that the lipids, proteins and RNAs of PDNs play a synergistic effect on microglia polarization and RNAs had the strongest anti-inflammation effect. Based on the result of RNA sequencing and KEGG analysis, we suggested that stu-miR156f-5p may be the functional element of PDN. Finally, WB assay demonstrated that PDN could alter microglia phenotype by downregulating autophagy-related proteins, and AMPK signaling pathway was involved in this process.

In this work, we demonstrated that PDNs decreased infarct volume, improved behavioral outcome and maintained BBB integrity. The multifaceted function was similar to that of PN, indicating that PDELNs can inherit the biological characters of its origin plants. For example, PNS, the major active component of PN, was reported to protect cerebral microvascular endothelial cells against oxygen–glucose deprivation/reperfusion induced barrier dysfunction [44]. In another study, XST, a PN injection, was reported to protect against ischemic stroke by modulating microglia phenotype [45]. And that is why we choose PN as the source of PDELNs for CI/R treatment in the study. Similar to our findings, Zhuang et al. [29] demonstrated that ginger-derived nanoparticles protected against alcohol-induced liver damage, which was similar to the biological effect of ginger. And the work emphasized that the activation of Nfr2 signaling pathway of ginger derived nanoparticles is dependent on 6-shogaol, which is a dehydrated analogues of the gingerols and enriched in ginger extract. So, it is recommended and reliable to select ELN sources according to the original biological activity of plants.

In our experimental design, XST was selected as a positive control, whose active component was PNS. Our results demonstrated that PDNs had a better therapeutic effect than XST. However, based on LC/MS analysis, PDN only contained nanogram scale of saponins Rg1, R1 and Rb1 per gram protein, which was much lower than that in XST. The better therapeutic outcome of PDN can be rationalized by two considerations. Firstly, PDNs contain not only saponins but also other functional molecules (lipids, proteins, nucleic acids and etc.), and they could play a synergistic effect to cure CI/R injury. Secondly, unlike free form of saponins, we supposed that saponins in PDN can be target-delivered into brain parenchymal and thus exert a strong and direct effect.

Besides the possible effect of saponins, we focused on the function of PDN miRNAs in this work. Briefly, PDN miRNAs were sequenced and their mimics were transfected into cells to analyze their biological functions. MiRNAs are among the most highly investigated species of the exosomal payload, and proved to be functional element of exosomes. To verify the function of exosomal miRNAs, target miRNAs were reported be knocked out from their origin cells. The resulting exosomes would be the same with natural exosomes except for the absence of particular miRNA. The biological effect of knock-out exosomes and natural exosomes were compared to testify the function of the miRNA, which was easy to implement when studying cell-derived exosomes. However, knocking out genes from plants was very difficult, which leads to the short for convenient and widely-accepted methods to study miRNAs from PDELNs.

Last but not the least, in this study we demonstrated that PDNs’ lipid might be responsible for its therapeutic effect. According to the lipidomic profile, more than 80% of the lipidomic components were found to be unsaturated lipids, which could exert anti-oxidant effect. Furthermore, abundant hydrophobic saponins might be another bioactive material from PDNs’ lipidomic components. Previous study has also proved the lipids to be therapeutically active from PDELNs. For example, lipids from grape exosome-like nanoparticles play of role in induction od Lgr5^+^ stem cells and in vivo targeting of intestinal stem cells [46]. In this study we only demonstrated the comprehensive effect of total lipids from PDNs, further studies are needed to elucidate the particular therapeutic effective component from PDN.

Biodistribution of exosomes or ELNs in the body is another heated topic. In this work, we demonstrated that PDNs could enter the brain parenchyma in a tMCAO model rat. More intriguingly, the accumulation of PDNs continued to increase in 24 h after injection, implying a retention of PDNs in the brain. Similarly, Gyeong et al. [47] reported that mesenchymal stem cell (MSC)-derived exosomes were found to enter the brain in a rat stroke model 3 h after injection. In another study, human cardiosphere-derived exosomes (labelled with DiD) were injected into an embolic stroke model rabbit. The brain was collected 24 h after administration and imaged. Based on the IVIS images, exosomes entered the brain parenchyma and accumulated in the ischemic hemisphere [48]. However, during the CI/R process, the BBB in the ischemic hemisphere was perished and its permeability went through significant decreased. Hence, exosomes might enter the brain via the leaky blood vessels rather than “cross” the BBB. However, PDN could be detected in both affected and contralateral brains, indicative of its ability to cross the BBB and potential to be a drug delivery system for other brain diseases. Moreover, in most published articles, exosomes were visualized in ex vivo brains at single time point, and little is known about their dynamic accumulation and elimination in the brain. Our work provided a real-time image of PDELNs’ in vivo behavior.

Overall, we isolated and characterized exosome-like nanoparticles from PN, and demonstrated that PDNs can alleviate I/R injury and improve behavioral outcome. To our knowledge, this is the first work investigating PDENLs’ effect in brain diseases. The results of the study showed that PDNs exert neuroprotective effect through inducing M2-like polarization in microglia. PDNs were identified to be a promising candidate for CI/R injury.

## Conclusions

In conclusion, our study explored PDNs as effective therapeutic and possible drug delivery system for treating CI/R injury. PDNs could enter the brain parenchyma without modification and altered the microglia polarization post reperfusion. Furthermore, lipids were the functional elements for PDNs’ effect towards CI/R injury.

### Supplementary Information

**Additional file 1: Figure S1.** Analysis of PDN lipidomic profile. **Figure S2**. Analysis of PDN proteomic profile. **Figure S3.** H&E staining of major organs from control group and PDNs treated group. **Figure S4.** Therapeutic effect of low, medium and high dose of PDNs. **Figure S5. **Whole blood cell analysis of rat after PDN injection. **Figure S6.** Top 20 of most enriched KEGG pathways of PDN miRNAs. **Table S1**. Lipids found in Panax notoginseng-derived exosome-like nanoparticles, as assessed by lipid profile analysis. **Table S2.** Proteins found in Panax notoginseng-derived exosome-like nanoparticles and subcellular localization, as assessed by proteomic analysis. **Table S3.** miRNAs from PDN.

## Data Availability

The data that support the findings of this study are available from the corresponding author, J.X. Wang, upon reasonable request.

## Abbreviations

PN: Panax notoginseng
PDNs: Panax notoginseng-derived exosome-like nanoparticles
I/R: Ischemia/reperfusion
tMCAO: Transient middle cerebral artery occlusion
BBB: Blood brain barrier
DiD: 1,1ʹ-Dioctadecyl-3,3,3ʹ,3ʹ -tetramethylindodicarbocyanine perchlorate
DiR: 1,1ʹ -Dioctadecyl-3,3,3ʹ,3ʹ -tetramethylindotricarbocyanine iodide
iv: Intravenously
XST: Xuesaitong
