# Supplementary material for: Panax notoginseng: derived exosome-like nanoparticles attenuate ischemia reperfusion injury via altering microglia polarization
Source: J Nanobiotechnology. 2023 Nov 10;21:416. doi: 10.1186/s12951-023-02161-1 (PMC10636993; doi:10.1186/s12951-023-02161-1)
Supplement: Supplementary file 1 — Additional file 1: Figure S1. Analysis of PDN lipidomic profile. Figure S2. Analysis of PDN proteomic profile. Figure S3. H&E staining of major organs from control group and PDNs treated group. Figure S4. Therapeutic effect of low, medium and high dose of PDNs. Figure S5. Whole blood cell analysis of rat after PDN injection. Figure S6. Top 20 of most enriched KEGG pathways of PDN miRNAs. Table S1. Lipids found in Panax notoginseng-derived exosome-like nanoparticles, as assessed by lipid profile analysis. Table S2. Proteins found in Panax notoginseng-derived exosome-like nanoparticles and subcellular localization, as assessed by proteomic analysis. Table S3. miRNAs from PDN. [file 12951_2023_2161_MOESM1_ESM.docx]

**Contents**

**Figure S1.** Analysis of PDN lipidomic profile.

**Figure S2**. Analysis of PDN proteomic profile.

**Figure S3.** H&E staining of major organs from control group and PDNs treated group

**Figure S4.** Therapeutic effect of low, medium and high dose of PDNs

**Figure S5.** Whole blood cell analysis of rat after PDN injection.

**Figure S6.** Top 20 of most enriched KEGG pathways of PDN miRNAs.

**Table S1**. Lipids found in Panax notoginseng-derived exosome-like nanoparticles, as assessed by lipid profile analysis.

**Table S2.** Proteins found in Panax notoginseng-derived exosome-like nanoparticles and subcellular localization, as assessed by proteomic analysis.

**Table S3.** miRNAs from PDN.

**
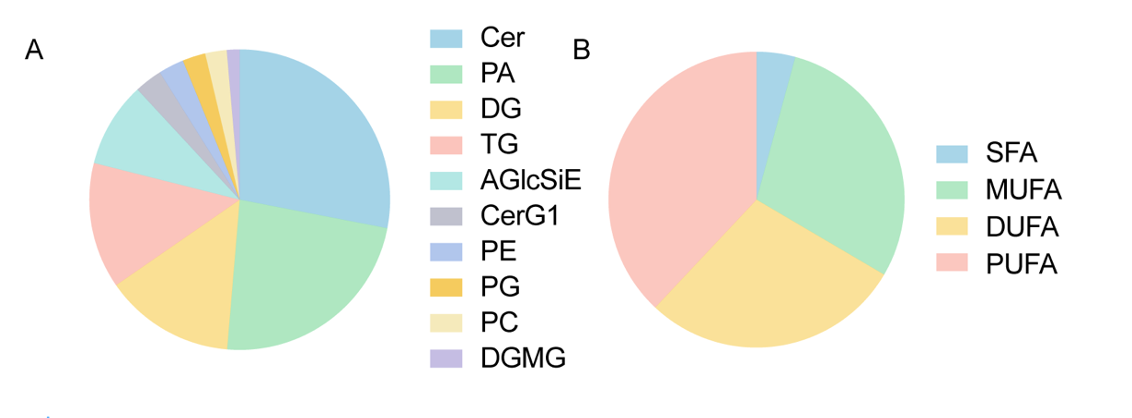
Figure S1.** Analysis of PDN lipidomic profile (A) Portions of top ten lipid subclass. (Cer, ceramides; PA, phosphatidic acid; DG, diacylglycerol; TG, triacylglycerol; AGlcSiE, acylated glucosyl betasitosterol ester; CerG1, glycosyl ceramide; PE, phosphatidylethanolamine; PG, phosphatidylglycerol; PC, phosphatidylcholine; DGMG, digalactosylmonoacylglycerol). (B) Portion of saturated fatty acid (SFA), monounsaturated fatty acid (MUFA), diunsaturated fatty acid (DUFA) and polyunsaturated fatty acid (PUFA) in PDN.

**Figure S2.** Analysis of PDN proteomic profile. (A) Cellular localization of PDN proteins. (B) Function of proteins detected in PDNs.


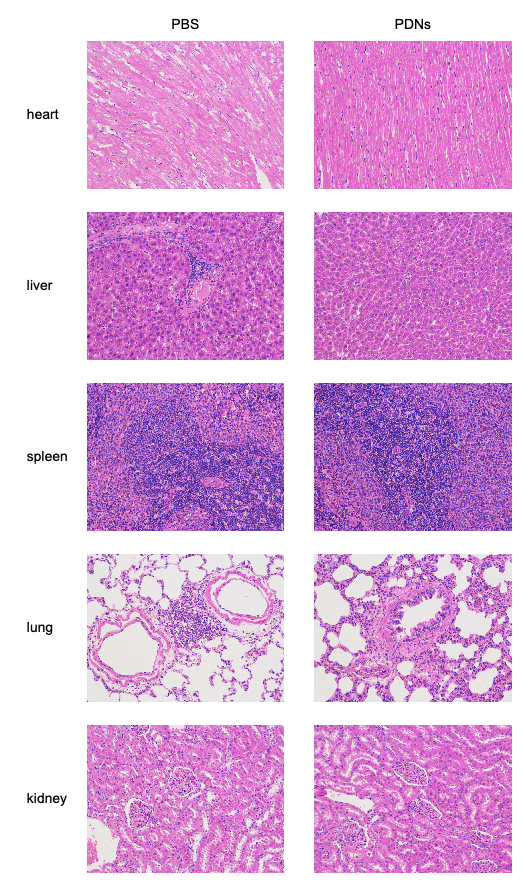


**Figure S3.** H&E staining of major organs from control group and PDNs treated group. For each group of rats, one representative image is shown.


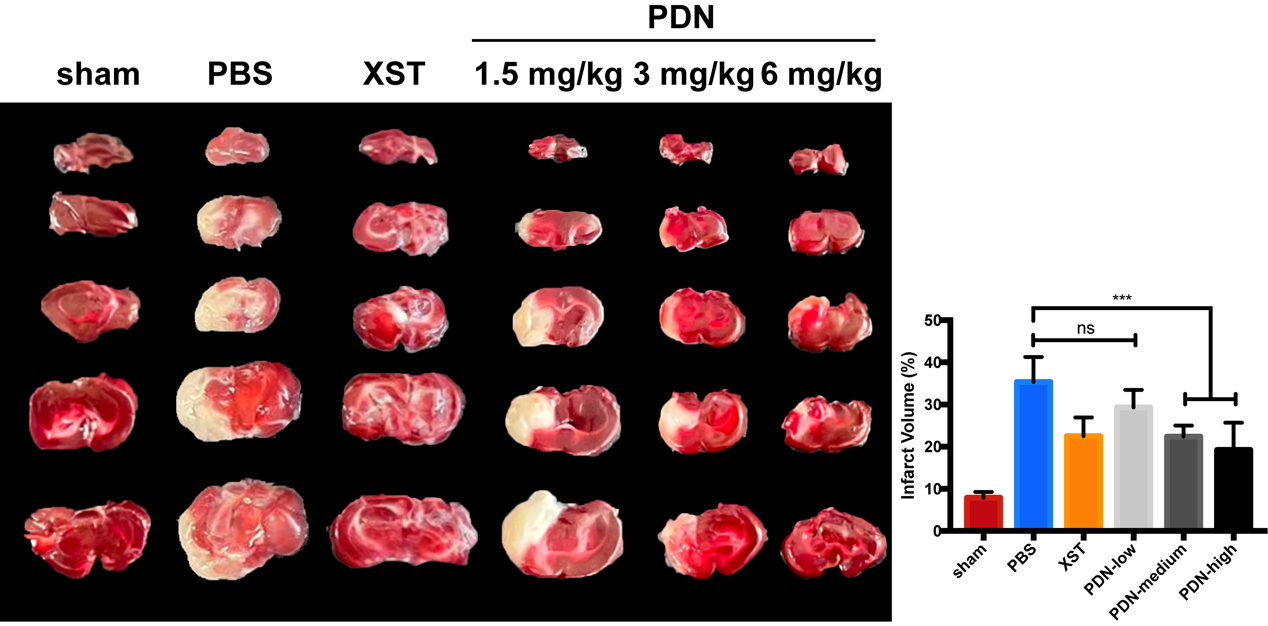


Fig S4. Therapeutic effect of low, medium and high dose of PDNs. Representative TTC staining and quantitative analysis of infarct volume of CI/R rats. (n=5)

**Figure S5.** Whole blood cell analysis of rat after PDN injection. HGB, haemoglobin; Lymph, lymphocyte; Gran, granule; MCHC, Mean corpuscular haemoglobin concentration; MCV, mean corpuscular volume; Mon, monocyte; MPV, mean platelet volume; PDW, platelet distribution width; PLT, platelets; RBC, red blood cell; RDW, red blood cell distribution width; WBC, white blood cell.

**Table S1.** Lipids found in Panax notohinseng-derived exosome-like nanopartices, as assessed by lipid profile analysis.

| LipidIon | % of total lipid |
| --- | --- |
| AGlcSiE(18:3)+H | 0.005902476 |
| AGlcSiE(20:0)+NH_4_ | 0.008860085 |
| AGlcSiE(25:0)+NH_4_ | 0.015291792 |
| AGlcSiE(20:1)+NH_4_ | 0.023334765 |
| AGlcSiE(23:0)+NH_4_ | 0.023550265 |
| AGlcSiE(17:1)+NH_4_ | 0.024143571 |
| AGlcSiE(18:2)+H | 0.0281218 |
| AGlcSiE(17:0)+_NH4_ | 0.039521801 |
| AGlcSiE(20:3)+NH_4_ | 0.041591765 |
| AGlcSiE(24:0)+NH_4_ | 0.048415281 |
| AGlcSiE(20:2)+NH_4_ | 0.051297069 |
| AGlcSiE(15:0)+NH_4_ | 0.055683023 |
| AGlcSiE(18:0)+NH_4_ | 0.141244303 |
| AGlcSiE(18:2)+NH_4_ | 0.157824663 |
| AGlcSiE(17:2)+NH_4_ | 0.189326381 |
| AGlcSiE(16:1)+NH_4_ | 0.23645584 |
| AGlcSiE(18:1)+NH_4_ | 0.519401317 |
| AGlcSiE(18:3)+NH_4_ | 1.007143337 |
| AGlcSiE(16:0)+NH_4_ | 1.229659788 |
| AGlcSiE(18:2)+NHNH_4_ | 4.786048427 |
| **Total AGlcSiE** | 8.632817749 |
| Cer(d52:3+pO)+HCOO | 0.001115112 |
| Cer(d33:0+pO+O)+Na | 0.001537355 |
| Cer(d18:1+hO/22:1)+HCOO | 0.001979697 |
| CerG1(d16:0+pO/18:2+O)+H | 0.002705879 |
| Cer(d17:1+hO/16:0)+HCOO | 0.002765641 |
| Cer(d17:1+hO/20:0+O)-H | 0.003196693 |
| Cer(d18:0+pO/27:0+O)+H | 0.003646757 |
| Cer(d18:2/16:1)+H | 0.003787152 |
| Cer(d34:2)+H | 0.003988996 |
| Cer(d18:1+hO/23:0)+H | 0.004296319 |
| Cer(d18:0+pO/26:0)+H | 0.004330307 |
| Cer(d18:2/24:0)+H | 0.004646178 |
| Cer(d25:0+pO/18:2+O)+H | 0.004735413 |
| Cer(d17:0+pO/22:0+O)-H | 0.004779583 |
| Cer(d46:1+hO+O)-H | 0.005115388 |
| Cer(d18:1+hO/26:0)-H | 0.005246256 |
| Cer(d26:0+pO)+H | 0.005473823 |
| Cer(d17:1+hO/18:0+O)-H | 0.005784445 |
| Cer(d36:0+pO+O)+H | 0.005817893 |
| CerG1(d43:3)+_4_ | 0.006016362 |
| CerG1(d34:3)-H | 0.006211928 |
| Cer(d50:7+hO)-H | 0.00643454 |
| Cer(d18:1+hO/25:0+O)+H | 0.006442907 |
| Cer(d18:1+hO/25:1)+H | 0.00658597 |
| Cer(d18:0+pO/26:0)+HCOO | 0.006865324 |
| Cer(d18:1+hO/27:0)+H | 0.006910976 |
| Cer(d18:1+hO/20:0)-H | 0.006953967 |
| Cer(d39:0+pO)+HCOO | 0.006981854 |
| Cer(d18:0+pO/18:0)+HCOO | 0.007090595 |
| Cer(d18:1+hO/16:0)-H | 0.007387222 |
| Cer(d37:1+pO+O)+HCOO | 0.007390561 |
| Cer(d17:1+hO/16:0+O)-H | 0.007728051 |
| Cer(d38:1+pO+O)+H | 0.007784845 |
| Cer(d18:2/22:0+O)+H | 0.007953229 |
| Cer(d18:1+hO/28:0+O)+H | 0.008189839 |
| Cer(d18:1+hO/26:0)+H | 0.008322289 |
| CerG1(d43:2+2O)+H | 0.008516325 |
| Cer(d18:1+hO/16:0)+HCOO | 0.009658968 |
| Cer(d18:2/23:0+O)+H | 0.010925723 |
| Cer(d38:0)+H | 0.011140579 |
| Cer(d18:1+hO/18:0)+HCOO | 0.011455992 |
| Cer(d18:1+hO/18:0)+H | 0.011487414 |
| Cer(d36:0)+H | 0.011546177 |
| Cer(d36:0+pO+O)+HCOO | 0.011696254 |
| Cer(d17:1+hO/28:0)+HCOO | 0.011750426 |
| Cer(d18:0+pO/16:0)+HCOO | 0.011849434 |
| Cer(d18:1+hO/25:0)-H | 0.01281975 |
| CerG1(d18:2/16:1)+H | 0.012936224 |
| Cer(d18:2/24:0+O)+H | 0.013593859 |
| Cer(d18:1+hO/25:0)+H | 0.013847554 |
| CerG1(d18:2/16:0+O)+H | 0.014468121 |
| Cer(d36:1+O)+H | 0.014553357 |
| Cer(d17:1+hO/16:0+O)+H | 0.015278716 |
| Cer(d17:0+pO/27:0+O)-H | 0.015641559 |
| Cer(d33:1+hO+O)+HCOO | 0.015841073 |
| Cer(d18:0+pO/23:0)+H | 0.016891837 |
| Cer(d41:1+hO)+HCOO | 0.017247438 |
| CerG1(d18:1+hO/23:0)+H | 0.017355755 |
| Cer(d17:1+hO/19:0+O)-H | 0.017892594 |
| Cer(d18:1+hO/22:0)-H | 0.018142407 |
| Cer(d18:1+hO/24:0)+H | 0.018910091 |
| Cer(d36:2+O)+H | 0.019359469 |
| Cer(d18:1+hO/21:0)+H | 0.019493386 |
| Cer(d18:2/26:0+O)+H | 0.019765833 |
| Cer(d18:2/25:0+O)+H | 0.020628881 |
| Cer(d18:2/16:0+O)+H | 0.020644552 |
| Cer(d18:1+hO/28:0+O)+HCOO | 0.021049627 |
| Cer(d18:1+hO/23:0)-H | 0.022375627 |
| CerG1(d18:1+hO/21:0+O)+H | 0.022493708 |
| Cer(d17:0+pO/26:0)+HCOO | 0.022809695 |
| Cer(d24:0/16:0)+HCOO | 0.023273025 |
| Cer(d17:0+pO/26:0+O)-H | 0.023929613 |
| Cer(d18:1+hO/27:0+O)+H | 0.024185372 |
| CerG1(d18:1+hO/22:0)+H | 0.024654761 |
| Cer(d32:0)+H | 0.024822904 |
| Cer(d17:0+pO/23:0)+HCOO | 0.025090773 |
| CerG1(d34:1+O)+H | 0.025374865 |
| Cer(d18:0+pO/23:0)+HCOO | 0.025563795 |
| CerG1(d17:1+hO/16:0+O)+H | 0.026656327 |
| Cer(d17:0+pO/17:0+O)-H | 0.0267053 |
| Cer(d38:0+pO+O)-H | 0.028071358 |
| CerG1(d53:7)+H | 0.028243655 |
| Cer(d18:0+pO/25:0)+H | 0.030680168 |
| CerG1(d18:1+hO/26:0)+H | 0.030877667 |
| Cer(d18:1+hO/20:0+O)+H | 0.03222976 |
| CerG1(d18:1+hO/18:0+O)+H | 0.034262313 |
| Cer(d18:2/24:0+O)+H | 0.036161468 |
| Cer(d18:0+pO/16:0)+H | 0.036999746 |
| CerG1(d45:5)+_4_ | 0.038061048 |
| Cer(d18:1+hO/24:0)-H | 0.038286166 |
| Cer(d34:2)+H | 0.039318292 |
| Cer(d17:1+hO/19:0+O)+HCOO | 0.040104629 |
| Cer(d18:0+pO/24:0)+HCOO | 0.04149725 |
| Cer(d17:1+hO/28:0+O)-H | 0.042570269 |
| Cer(d18:1+hO/18:0+O)+H | 0.043161993 |
| Cer(d17:0/16:1)+H | 0.045801035 |
| Cer(d18:1+hO/21:0)+HCOO | 0.045862043 |
| Cer(d18:0+pO/26:0+O)+H | 0.046232979 |
| Cer(d17:0+pO/17:0+O)+HCOO | 0.047962964 |
| Cer(d42:1+hO)+HCOO | 0.049943246 |
| Cer(d17:0+pO/25:0+O)-H | 0.051935668 |
| Cer(d18:0+pO/22:0+O)+H | 0.054572718 |
| Cer(d42:2+hO+O)+HCOO | 0.054590692 |
| CerG1(d18:1+hO/25:0)+H | 0.055386886 |
| Cer(d18:1/16:0+O)+H | 0.055516854 |
| Cer(d18:1+hO/20:0+O)+H | 0.057503486 |
| CerG1(d18:1+hO/20:0+O)+H | 0.063516097 |
| Cer(d18:1+hO/16:0)+HCOO | 0.071873573 |
| Cer(d18:0+pO/23:0+O)+H | 0.072972821 |
| Cer(d18:0+pO/20:0+O)+HCOO | 0.073617589 |
| Cer(d18:1+hO/20:0)+HCOO | 0.077106798 |
| Cer(d17:0+pO/24:0+O)+HCOO | 0.077404803 |
| Cer(d38:1+hO+O)-H | 0.078417414 |
| CerG1(d18:1+hO/24:0)+H | 0.078917294 |
| Cer(d18:1+hO/25:0+O)+H | 0.081751929 |
| Cer(d20:0+pO/20:0+O)+HCOO | 0.082183577 |
| Cer(d17:0+pO/26:0+O)+HCOO | 0.08345876 |
| Cer(d18:1+hO/26:0)+HCOO | 0.085272508 |
| CerG1(d42:1+hO)+H | 0.088324504 |
| Cer(d18:1+hO/27:0+O)+H | 0.113020955 |
| Cer(d18:1+hO/21:0+O)+H | 0.115745808 |
| Cer(d18:1+hO/26:0)+H | 0.11966802 |
| Cer(d18:1+hO/16:0+O)+H | 0.122816207 |
| CerG1(d18:1/16:0+O)+H | 0.125820343 |
| CerG1(d18:1+hO/23:0+O)+H | 0.131522652 |
| Cer(d18:1+hO/22:0)+H | 0.134002237 |
| Cer(d18:0+pO/24:0)+H | 0.154456526 |
| Cer(d17:1+hO/28:0+O)+HCOO | 0.16560219 |
| CerG1(d18:1+hO/22:0+O)+H | 0.169096091 |
| CerG1(d18:1+hO/25:0+O)+H | 0.169283076 |
| Cer(d17:0+pO/25:0+O)+HCOO | 0.172413906 |
| Cer(d18:1+hO/24:0+O)+H | 0.172493989 |
| Cer(d17:1+hO/27:0+O)-H | 0.189951399 |
| Cer(d38:1+pO+O)+HCOO | 0.191588235 |
| Cer(d18:0+pO/24:0+O)+H | 0.19210148 |
| Cer(d18:1+hO/25:0)+HCOO | 0.207164667 |
| Cer(d18:1+hO/23:0)+H | 0.224853816 |
| Cer(d18:0+pO/25:0+O)+H | 0.234397303 |
| Cer(d18:1+hO/22:0)+HCOO | 0.235319155 |
| Cer(d39:1+hO+O)-H | 0.242266953 |
| CerG1(d32:3+hO)-H | 0.267761083 |
| CerG1(d18:1+hO/26:0+O)+H | 0.271753171 |
| Cer(d18:1+hO/16:0+O)+H | 0.288896466 |
| Cer(d18:1+hO/23:0)+HCOO | 0.315442878 |
| Cer(d17:1+hO/17:0+O)-H | 0.329612797 |
| Cer(d40:1+hO+O)-H | 0.352974099 |
| CerG1(d18:1+hO/24:0+O)+H | 0.411800138 |
| Cer(d18:1+hO/24:0)+H | 0.421247114 |
| Cer(d18:1+hO/25:0+O)-H | 0.422446398 |
| Cer(d17:1+hO/24:0+O)-H | 0.453644729 |
| Cer(d18:1+hO/22:0+O)+H | 0.496760944 |
| Cer(d18:1+hO/24:0)+HCOO | 0.577460011 |
| Cer(d17:1+hO/17:0+O)+HCOO | 0.622715168 |
| Cer(d18:1+hO/21:0+O)+HCOO | 0.649804623 |
| CerG1(d18:1+hO/16:0+O)+H | 0.678548209 |
| Cer(d17:1+hO/27:0+O)+HCOO | 0.689270624 |
| Cer(d18:1+hO/23:0+O)+H | 0.816177401 |
| Cer(d18:1+hO/25:0)+H | 0.838222829 |
| Cer(d17:1+hO/25:0+O)-H | 0.882278472 |
| Cer(d40:1+pO+O)+HCOO | 1.106612543 |
| Cer(d17:1+hO/24:0+O)+HCOO | 1.407402093 |
| Cer(d18:1+hO/25:0+O)+HCOO | 1.456780491 |
| Cer(d18:1+hO/25:0+O)+H | 1.616092777 |
| Cer(d18:1+hO/26:0+O)+H | 1.685220831 |
| Cer(d18:1+hO/24:0+O)+H | 2.575548126 |
| Cer(d42:1+pO+O)+HCOO | 2.883010222 |
| **Total Cer** | 26.39168127 |
| **Total CerG1** | 2.810564481 |
| CerG2(d30:3)+NH_4_ | 0.001225908 |
| CerG2(d31:2)-H | 0.086835483 |
| CerG2(d32:3+hO)-H | 0.473039823 |
| **Total CerG2** | 0.561101214 |
| CerG3GNAc1(d30:0+pO)-H | 0.002830776 |
| CerG3GNAc1(d43:2+pO)-H | 0.005698989 |
| **Total CerGNAc1** | 0.008529765 |
| CerP(d18:1+hO/25:0+O)+H | 0.191034038 |
| CerP(d18:1+hO/16:0+O)+H | 0.018099968 |
| CerP(d18:1+hO/20:0+O)+H | 0.011857957 |
| CerP(d27:1)+H | 0.002748336 |
| CerP(d18:1+hO/24:0+O)+H | 0.160459609 |
| CerP(d18:1+hO/25:0+O)+H | 0.064328028 |
| **Total CerP** | 0.448527936 |
| CL(18:2/16:0/16:0/16:0)-H | 0.001534912 |
| CL(18:2/16:0/18:2/16:0)-H | 0.004992446 |
| CL(18:2/16:0/16:0/18:1)-H | 0.001088792 |
| CL(20:5/16:0/16:0/18:2)-H | 0.000982139 |
| CL(18:2/16:0/18:2/18:2)-H | 0.006129019 |
| CL(18:2/16:0/18:1/18:2)-H | 0.001295687 |
| CL(18:4/18:2/18:2/18:2)-H | 0.00377302 |
| CL(18:3/18:2/18:2/18:2)-H | 0.001042767 |
| CL(18:3/18:2/18:2/18:2)-H | 0.00422246 |
| CL(18:2/18:2/18:2/18:2)-H | 0.057766257 |
| CL(18:2/18:1/18:2/18:2)-H | 0.008404587 |
| CL(20:5/18:2/18:2/18:2)-H | 0.010661187 |
| CL(18:2/18:2/18:2/20:4)-H | 0.00165127 |
| CL(81:10)-H | 0.00256934 |
| CL(83:13)-H | 0.001311812 |
| CL(83:12)-H | 0.008367451 |
| CL(83:12)-H | 0.002552739 |
| **Total CL** | 0.118345883 |
| Co(Q9)+H | 0.098870239 |
| Co(Q9)+NH_4_ | 0.128809714 |
| Co(Q10)+H | 0.017978562 |
| Co(Q10)+NH_4_ | 0.031085556 |
| **Total Co** | 0.276744071 |
| DG(18:3/18:2)+NH_4_ | 0.147034155 |
| DG(18:3/18:2)+NH_4_ | 0.002748888 |
| DG(18:2/18:2)+NH_4_ | 0.002942679 |
| DG(18:2/18:2)+NH_4_ | 1.03750642 |
| DG(18:2/18:2)+NH_4_ | 0.102191125 |
| DG(18:2/18:2)+NH_4_ | 0.011254447 |
| DG(18:1/18:2)+NH_4_ | 0.015511725 |
| DG(18:1/18:2)+NH_4_ | 0.159333651 |
| DG(18:1/18:1)+NH_4_ | 0.046837576 |
| DG(20:2/18:2)+NH_4_ | 0.05621028 |
| DG(20:1/18:2)+NH_4_ | 0.009614145 |
| DG(38:3)+Na | 0.002419072 |
| DG(18:2/23:0)+NH_4_ | 0.006857552 |
| DG(16:0/12:3)+H | 0.00216769 |
| DG(24:0/18:2)+NH_4_ | 0.012321655 |
| DG(25:0/18:2)+NH_4_ | 0.00517582 |
| DG(32:1p)+Na | 0.013541932 |
| DG(15:0/18:3)+H | 0.001332786 |
| DG(18:4/16:0)+H | 0.082159065 |
| DG(16:0/18:3)+H | 0.057616753 |
| DG(16:0/18:3)+H | 0.011675053 |
| DG(16:0/18:2)+H | 0.045547309 |
| DG(16:0/18:2)+H | 0.008108865 |
| DG(15:0/18:2)+NH_4_ | 0.018176058 |
| DG(34:3p)+Na | 0.007355555 |
| DG(34:3p)+Na | 0.018851117 |
| DG(34:3p)+Na | 0.002499425 |
| DG(34:2p)+Na | 0.062997086 |
| DG(34:2p)+Na | 0.031301375 |
| DG(33:2)+Na | 0.044669776 |
| DG(18:4/16:1)+NH_4_ | 0.003678814 |
| DG(18:4/16:0)+NH_4_ | 0.454514156 |
| DG(16:0/18:3)+NH_4_ | 0.013721055 |
| DG(16:0/18:3)+NH_4_ | 0.069914586 |
| DG(16:0/18:2)+NH_4_ | 1.600933193 |
| DG(16:0/18:2)+NH_4_ | 0.00374564 |
| DG(16:0/18:2)+NH_4_ | 0.008677507 |
| DG(16:0/18:1)+NH_4_ | 0.017029226 |
| DG(18:3/18:3)+H | 0.014603987 |
| DG(18:3/18:3)+H | 0.013799272 |
| DG(18:3/18:3)+H | 0.004359648 |
| DG(18:3/18:3)+H | 0.003663989 |
| DG(18:3/18:2)+H | 0.041365712 |
| DG(18:3/18:2)+H | 0.02075052 |
| DG(18:3/18:2)+H | 0.028377066 |
| DG(18:2/18:2)+H | 0.102156547 |
| DG(18:2/18:2)+H | 0.123187446 |
| DG(18:1/18:3)+H | 0.032530407 |
| DG(18:1/18:3)+H | 0.006187831 |
| DG(18:1/18:2)+H | 0.03577519 |
| DG(35:3)+NH_4_ | 0.003962273 |
| DG(17:0/18:2)+NH_4_ | 0.026127605 |
| DG(18:3/18:3)+NH_4_ | 0.094903052 |
| **Total DG** | 13.19755046 |
| DGDG(18:2/18:3)+Na | 0.044954655 |
| DGDG(16:0/18:2)+HCOO | 0.118710866 |
| DGDG(16:0/18:1)+HCOO | 0.0255004 |
| DGDG(18:2/18:3)+HCOO | 0.045898024 |
| DGDG(18:2/18:2)+HCOO | 0.214857973 |
| DGDG(18:0/18:2)+HCOO | 0.032344033 |
| DGDG(15:0/18:2)+Na | 0.002448627 |
| DGDG(16:0/18:3)+Na | 0.007296407 |
| DGDG(16:0/18:2)+Na | 0.17167756 |
| DGDG(16:0/18:1)+Na | 0.088824041 |
| DGDG(17:0/18:2)+Na | 0.017524959 |
| DGDG(18:3/18:3)+Na | 0.003657494 |
| DGDG(18:2/18:2)+Na | 0.205212989 |
| DGDG(18:1/18:2)+Na | 0.093243927 |
| DGDG(18:1/18:1)+Na | 0.073122763 |
| DGDG(18:3/20:2)+Na | 0.000396659 |
| DGDG(18:2/20:2)+Na | 0.011037446 |
| DGDG(16:0/18:2)-H | 0.003059074 |
| DGDG(16:0/16:0)+HCOO | 0.005864021 |
| DGDG(16:0/18:3)+HCOO | 0.00713332 |
| DGDG(17:0/18:2)+HCOO | 0.005743187 |
| DGDG(18:3/18:3)+HCOO | 0.006258537 |
| DGDG(18:1/18:2)+HCOO | 0.065973612 |
| DGDG(41:11)-H | 0.030315852 |
| DGDG(18:2/20:1)+HCOO | 0.004069813 |
| DGMG(16:0)+HCOO | 0.00188158 |
| DGMG(18:2)+HCOO | 0.008186952 |
| **Total DGMG** | 1.29519477 |
| LPA(18:2)-H | 0.196011134 |
| LPA(18:2)+NH_4_ | 0.011332259 |
| **Total LPA** | 0.207343394 |
| LPC(16:1)+H | 0.000564951 |
| LPC(16:0)+H | 0.015989435 |
| LPC(18:2)+H | 0.018334429 |
| LPC(18:1)+H | 0.001442806 |
| LPC(18:0)+H | 0.002954174 |
| LPC(20:5)+H | 0.002369986 |
| LPC(18:1)+Na | 0.001970807 |
| LPC(22:0)+H | 0.00108605 |
| LPC(16:0)+HCOO | 0.007570782 |
| LPC(18:2)+HCOO | 0.00981216 |
| LPC(18:0)+HCOO | 0.001232687 |
| LPC(24:2)+H | 0.062128003 |
| LPC(24:0)+H | 0.001108048 |
| **Total LPC** | 0.126564318 |
| LPE(16:0)+H | 0.005913082 |
| LPE(18:2)+H | 0.004531605 |
| LPE(22:0)+H | 0.001829951 |
| LPE(24:0)+H | 0.002020957 |
| LPE(16:0)-H | 0.006532271 |
| LPE(18:2)-H | 0.005849958 |
| LPE(18:0)-H | 0.000287473 |
| LPE(22:0)-H | 0.001295654 |
| LPE(23:0)-H | 0.001170794 |
| **Total LPE** | 0.029431745 |
| LPG(16:0)-H | 0.008236685 |
| **Total LPG** | 0.008236685 |
| MG(18:2)+H | 0.003771417 |
| MG(16:0)+H | 0.010121391 |
| **Total MG** | 0.013892808 |
| MGDG(18:2/18:3)+HCOO | 0.168342095 |
| MGDG(18:2/18:2)+HCOO | 0.460980117 |
| MGDG(15:1/18:2)+Na | 0.004147786 |
| MGDG(16:2/18:3)+Na | 0.002162872 |
| MGDG(16:2/18:2)+Na | 0.007839547 |
| MGDG(16:0/18:2)+Na | 0.194825124 |
| MGDG(16:0/18:1)+Na | 0.037564728 |
| MGDG(17:1/18:2)+Na | 0.007047649 |
| MGDG(17:0/18:2)+Na | 0.005521193 |
| MGDG(18:3/18:3)+Na | 0.108094669 |
| MGDG(18:2/18:3)+Na | 0.238373849 |
| MGDG(18:2/18:2)+Na | 0.678688446 |
| MGDG(18:1/18:2)+Na | 0.39216766 |
| MGDG(18:1/18:1)+Na | 0.031023444 |
| MGDG(18:0/18:1)+Na | 0.002368939 |
| MGDG(18:2/20:2)+Na | 0.014581127 |
| MGDG(18:2/20:1)+Na | 0.011848012 |
| MGDG(29:0)-H | 0.00314795 |
| MGDG(18:2/18:3)-H | 0.013758074 |
| MGDG(18:2/18:2)-H | 0.108611158 |
| MGDG(16:2/18:3)+HCOO | 0.004112727 |
| MGDG(16:1/18:2)+HCOO | 0.005888826 |
| MGDG(16:0/18:2)+HCOO | 0.061112602 |
| MGDG(16:0/18:1)+HCOO | 0.020890236 |
| MGDG(17:0/18:2)+HCOO | 0.00419323 |
| MGDG(18:3/18:3)+HCOO | 0.095150104 |
| MGDG(18:2/18:3)+HCOO | 0.01343067 |
| MGDG(18:1/18:2)+HCOO | 0.126220304 |
| MGDG(18:1/18:1)+HCOO | 0.01417124 |
| MGDG(18:2/20:2)+HCOO | 0.005719802 |
| **Total MGDG** | 2.841984181 |
| MGMG(16:0)+HCOO | 0.001727098 |
| MGMG(18:3)+HCOO | 0.002786956 |
| MGMG(18:2)+HCOO | 0.014475754 |
| **Total MGMG** | 0.018989808 |
| PA(36:4)+Na | 0.212995898 |
| PA(18:4/16:0)-H | 0.129838044 |
| PA(18:3/18:2)-H | 0.134130926 |
| PA(34:3)+H | 0.034869883 |
| PA(34:2)+H | 0.357810517 |
| PA(34:2)+H | 0.066182619 |
| PA(34:2)+H | 0.131467719 |
| PA(15:0/18:2)+NH_4_ | 0.031349138 |
| PA(16:0/18:3)+NH_4_ | 0.022429962 |
| PA(16:0/18:3)+NH_4_ | 0.052918466 |
| PA(16:0/18:3)+NH_4_ | 0.037206743 |
| PA(16:0/18:2)+NH_4_ | 0.215492871 |
| PA(16:0/18:2)+NH_4_ | 1.673107151 |
| PA(16:0/18:2)+NH_4_ | 0.205820964 |
| PA(16:0/18:2)+NH_4_ | 0.125675506 |
| PA(16:0/18:2)+NH_4_ | 0.486417438 |
| PA(36:5)+H | 0.110763422 |
| PA(34:2)+Na | 0.113120072 |
| PA(34:2)+Na | 0.330123144 |
| PA(17:0/18:2)+NH_4_ | 0.026325108 |
| PA(18:2/18:2)+NH_4_ | 0.549303797 |
| PA(18:2/18:2)+NH_4_ | 2.969443616 |
| PA(18:2/18:2)+NH_4_ | 0.097947172 |
| PA(18:2/18:2)+NH_4_ | 0.085681738 |
| PA(18:2/18:2)+NH_4_ | 0.065206946 |
| PA(18:2/18:2)+NH_4_ | 0.033990072 |
| PA(18:2/18:2)+NH_4_ | 0.063735047 |
| PA(18:2/18:2)+NH_4_ | 0.123941194 |
| PA(18:2/18:2)+NH_4_ | 0.020492615 |
| PA(18:2/18:2)+NH_4_ | 0.054139331 |
| PA(18:0/18:2)+NH_4_ | 0.134758543 |
| PA(36:4)+Na | 0.285409209 |
| PA(20:1/18:2)+NH_4_ | 0.015687299 |
| PA(24:0/18:2)+NH_4_ | 0.023490546 |
| PA(15:0/18:2)-H | 0.084222037 |
| PA(16:0/18:3)-H | 0.060527713 |
| PA(16:0/18:3)-H | 0.088942386 |
| PA(16:0/18:2)-H | 7.132808681 |
| PA(16:0/18:1)-H | 0.056433415 |
| PA(17:0/18:2)-H | 0.082137361 |
| PA(17:0/18:2)-H | 0.107656239 |
| PA(35:1)-H | 0.006243881 |
| PA(18:2/18:2)-H | 0.190775064 |
| PA(18:2/18:2)-H | 4.872720291 |
| PA(18:0/18:2)-H | 0.176657272 |
| PA(37:3)-H | 0.007398263 |
| PA(37:2)-H | 0.009039741 |
| PA(20:0/18:2)-H | 0.006433876 |
| PA(47:4)-H | 0.014061008 |
| **Total PA** | 21.91732995 |
| PC(33:2)+H | 0.005986146 |
| PC(34:3)+H | 0.014937719 |
| PC(34:2)+H | 0.831825845 |
| PC(34:1)+H | 0.147342648 |
| PC(36:5)+H | 0.006469562 |
| PC(36:4)+H | 0.178954096 |
| PC(36:4)+H | 0.06923326 |
| PC(36:3)+H | 0.104321189 |
| PC(36:2)+H | 0.124260086 |
| PC(38:4)+H | 0.009526111 |
| PC(40:3)+H | 0.023541859 |
| PC(16:0/16:1)+H | 0.029839464 |
| PC(32:0)+H | 0.02734891 |
| PC(33:3)+H | 0.003833159 |
| PC(33:1)+H | 0.011562398 |
| PC(34:5)+H | 0.001397948 |
| PC(34:4)+H | 0.027506604 |
| PC(34:2)+H | 0.024774445 |
| PC(19:1/16:0)+H | 0.026041259 |
| PC(34:2)+Na | 0.091724333 |
| PC(36:4)+H | 0.012528886 |
| PC(36:1)+H | 0.014505571 |
| PC(35:3)+Na | 0.035096641 |
| PC(37:2)+H | 0.016831121 |
| PC(18:2/18:2)+Na | 0.02616113 |
| PC(36:3)+Na | 0.011442555 |
| PC(18:1/18:1)+Na | 0.023056316 |
| PC(38:4)+H | 0.026330963 |
| PC(38:2)+H | 0.035355333 |
| PC(39:3)+H | 0.002345904 |
| PC(41:2p)+H | 0.03289419 |
| PC(40:2)+H | 0.024821749 |
| PC(41:2)+H | 0.027838077 |
| PC(22:0/18:2)+Na | 0.002480405 |
| PC(42:3)+H | 0.040805809 |
| PC(42:2)+H | 0.054706718 |
| PC(42:2)+H | 0.008504264 |
| PC(43:3)+H | 0.016905989 |
| PC(24:0/18:2)+Na | 0.003983702 |
| PC(44:2)+H | 0.003864432 |
| **Total PC** | 2.180886795 |
| PE(34:2)+H | 0.418120367 |
| PE(18:2/18:2)+H | 0.03953502 |
| PE(40:4)+H | 0.39090493 |
| PE(41:2)+H | 0.04423632 |
| PE(42:2)+H | 0.097867548 |
| PE(16:0/18:2)-H | 0.120629705 |
| PE(18:2/18:2)-H | 0.028837034 |
| PE(18:0/18:2)-H | 0.008615082 |
| PE(20:2/18:2)-H | 0.003791196 |
| PE(18:0/20:0)-H | 0.371490948 |
| PE(22:0/18:2)-H | 0.013446601 |
| PE(18:2/23:0)-H | 0.017465352 |
| PE(24:1/18:2)-H | 0.021445731 |
| PE(24:0/18:2)-H | 0.026405065 |
| PE(33:1)+H | 0.019181007 |
| PE(33:1)+H | 0.033866862 |
| PE(34:2)+H | 0.348125722 |
| PE(34:1)+H | 0.098348151 |
| PE(17:1/18:2)+H | 0.010756177 |
| PE(35:2)+H | 0.010804097 |
| PE(36:6)+H | 0.000654893 |
| PE(34:2)+Na | 0.014106531 |
| PE(18:2/18:2)+H | 0.017653461 |
| PE(24:0/12:3)+H | 0.100191886 |
| PE(18:2/18:2)+Na | 0.002497051 |
| PE(36:4)+Na | 0.002638904 |
| PE(38:4)+H | 0.004912283 |
| PE(38:3)+H | 0.006586151 |
| PE(38:2)+H | 0.004548599 |
| PE(40:2)+H | 0.017061329 |
| PE(23:0e)+H | 0.001084706 |
| PE(43:2)+H | 0.021802691 |
| PE(16:0/16:1)-H | 0.00217247 |
| PE(16:0/18:3)-H | 0.002632596 |
| PE(16:0/18:2)-H | 0.007164501 |
| PE(16:0/18:1)-H | 0.003086173 |
| PE(17:0/18:2)-H | 0.004812594 |
| PE(18:3/18:2)-H | 0.000709981 |
| PE(18:2/18:2)-H | 0.052601381 |
| PE(18:1/18:2)-H | 0.002861133 |
| PE(18:1/18:2)-H | 0.005604985 |
| PE(16:0/20:1)-H | 0.001271552 |
| PE(36:0)-H | 0.13345814 |
| PE(20:1/18:2)-H | 0.004971853 |
| PE(20:0/18:2)-H | 0.003286732 |
| PE(18:2/21:1)-H | 0.00676348 |
| PE(18:2/21:0)-H | 0.002519017 |
| PE(22:1/18:2)-H | 0.011426073 |
| PE(25:1/18:2)-H | 0.009869245 |
| PE(25:0/18:2)-H | 0.009118001 |
| PE(26:0/18:2)-H | 0.001884915 |
| **Total PE** | 2.583826224 |
| PG(16:0/18:2)+NH_4_ | 0.024120764 |
| PG(16:0/16:0)-H | 0.084514943 |
| PG(16:0/18:2)-H | 0.100502329 |
| PG(16:0/18:1)-H | 0.061329256 |
| PG(18:0/16:0)-H | 0.018320174 |
| PG(36:7)-H | 0.4248642 |
| PG(32:0)+Na | 0.005298325 |
| PG(16:0/18:2)+H | 0.016179715 |
| PG(16:0/18:3)+NH_4_ | 0.000989859 |
| PG(16:0/18:1)+NH_4_ | 0.00813646 |
| PG(34:2)+Na | 0.007536987 |
| PG(18:2/18:2)+H | 0.001674499 |
| PG(18:2/18:2)+NH_4_ | 0.00434828 |
| PG(46:2)+H | 0.0644086 |
| PG(46:2)+H | 0.013433199 |
| PG(48:1)+H | 0.003993315 |
| PG(16:0/16:1)-H | 0.000666758 |
| PG(17:1/16:0)-H | 0.000627769 |
| PG(16:0/17:0)-H | 0.006433573 |
| PG(34:5)-H | 1.298150973 |
| PG(34:5)-H | 0.014435616 |
| PG(34:5)-H | 0.037964524 |
| PG(34:5)-H | 0.021607161 |
| PG(16:0/18:3)-H | 0.003181503 |
| PG(36:7)-H | 0.021130031 |
| PG(18:2/18:2)-H | 0.013995196 |
| PG(18:2/18:2)-H | 0.005901824 |
| PG(18:1/18:2)-H | 0.004700164 |
| PG(36:2)-H | 0.028363793 |
| PG(16:0/20:1)-H | 0.003272443 |
| PG(26:0e)+H | 0.021732697 |
| **Total PG** | 2.321814931 |
| PI(16:0/18:2)+H | 0.004323978 |
| PI(34:2)+NH_4_ | 0.009354064 |
| PI(16:0/18:2)-H | 0.04430976 |
| PI(16:0/18:2)+H | 0.041178546 |
| PI(34:2)+NH_4_ | 0.070076173 |
| PI(34:2)+Na | 0.004867338 |
| PI(36:4)+NH_4_ | 0.003261916 |
| PI(17:0/22:1)+NH_4_ | 0.015347279 |
| PI(18:2/18:2)-H | 0.001039091 |
| **Total PI** | 0.193758146 |
| PIP(54:6)-H | 0.004905821 |
| **Total PIP** | 0.004905821 |
| PS(45:3)+Na | 0.109681711 |
| PS(47:5)+Na | 0.167942698 |
| PS(36:1)-H | 0.002622728 |
| PS(37:1)-H | 0.171244256 |
| PS(37:0)-H | 0.014449868 |
| PS(39:3)-H | 0.053169191 |
| PS(18:2/21:0)-H | 0.019314646 |
| PS(39:1)-H | 0.029785216 |
| PS(16:0/16:0)+H | 0.01087367 |
| PS(34:2)+H | 0.004086323 |
| PS(22:0/18:2)+H | 0.020823222 |
| PS(18:2/23:0)+H | 0.016426609 |
| PS(24:0/18:2)+H | 0.022779954 |
| PS(43:1)+Na | 0.018082872 |
| PS(45:4)+Na | 0.006022069 |
| PS(45:2)+Na | 0.070007094 |
| PS(45:1)+Na | 0.01084623 |
| PS(46:3)+Na | 0.008200864 |
| PS(47:4)+Na | 0.067035455 |
| PS(47:3)+Na | 0.030107523 |
| PS(18:2/18:2)-H | 0.008677335 |
| PS(18:2/18:2)-H | 0.001833644 |
| PS(36:2)-H | 0.002087163 |
| PS(20:0/18:2)-H | 0.001885297 |
| PS(18:2/21:0)-H | 0.00145818 |
| PS(22:0/18:2)-H | 0.020364864 |
| PS(18:2/23:0)-H | 0.017567037 |
| PS(41:1)-H | 0.005944401 |
| PS(42:4)-H | 0.001472111 |
| PS(24:0/18:2)-H | 0.018079344 |
| PS(25:0/18:2)-H | 0.004125966 |
| PS(43:1)-H | 0.007269138 |
| **Total PS** | 0.944266681 |
| So(d18:1+hO)+H | 0.084238392 |
| So(d16:1)+H | 0.008598222 |
| So(d16:0+pO)+H | 0.018605865 |
| So(d18:1)+H | 0.002920607 |
| So(d18:1+hO)+H | 0.006542287 |
| So(d18:1+hO)+H | 0.007244389 |
| **Total So** | 0.128149763 |
| SQDG(16:0/16:0)-H | 0.010744304 |
| SQDG(34:3)-H | 0.002166876 |
| SQDG(34:2)-H | 0.037445344 |
| SQDG(34:1)-H | 0.02468183 |
| SQDG(18:2/18:3)-H | 0.002450237 |
| SQDG(18:2/18:2)-H | 0.011853746 |
| SQDG(36:3)-H | 0.007945789 |
| SQDG(36:2)-H | 0.01138338 |
| **Total SQDG** | 0.108671507 |
| TG(16:0/18:1/18:3)+NH_4_ | 1.557203634 |
| TG(18:2/18:2/18:2)+NH_4_ | 2.621839559 |
| TG(18:1/18:2/18:2)+NH_4_ | 0.922481884 |
| TG(18:3/18:2/18:2)+Na | 0.487825994 |
| TG(18:2/18:2/18:2)+Na | 0.90619701 |
| TG(8:0/10:2/18:2)+H | 0.202056225 |
| TG(8:0/10:2/18:2)+H | 0.011761747 |
| TG(6:0/12:2/18:2)+H | 0.002221803 |
| TG(8:0/10:2/18:2)+NH_4_ | 0.008769957 |
| TG(36:3)+NH_4_ | 0.008957585 |
| TG(6:0/12:2/18:0)+NH_4_ | 0.019470449 |
| TG(36:1)+NH_4_ | 0.047360465 |
| TG(6:0/12:2/20:2)+NH_4_ | 0.007211405 |
| TG(38:4)+NH_4_ | 0.011319506 |
| TG(6:0/14:0/18:3)+NH_4_ | 0.025143619 |
| TG(38:3)+NH_4_ | 0.005481948 |
| TG(38:2)+NH_4_ | 0.004579344 |
| TG(41:11)+H | 0.007036117 |
| TG(16:0/14:0/16:0)+NH_4_ | 0.001598253 |
| TG(16:0/14:2/16:0)+Na | 0.011150528 |
| TG(49:3p)+NH_4_ | 0.001415049 |
| TG(16:0/14:0/18:2)+NH_4_ | 0.00435466 |
| TG(16:0/14:0/18:1)+NH_4_ | 0.00351271 |
| TG(16:0/16:0/16:0)+NH_4_ | 0.004882582 |
| TG(18:2/13:0/18:2)+NH_4_ | 0.000580496 |
| TG(15:0/16:1/18:2)+NH_4_ | 0.003789961 |
| TG(15:0/16:0/18:2)+NH_4_ | 0.00860228 |
| TG(15:0/16:0/18:1)+NH_4_ | 0.002774667 |
| TG(14:0/18:2/18:3)+NH_4_ | 0.001913417 |
| TG(14:0/18:2/18:2)+NH_4_ | 0.015921861 |
| TG(16:0/16:0/18:3)+NH_4_ | 0.011820334 |
| TG(16:0/16:1/18:2)+NH_4_ | 0.001615683 |
| TG(16:0/16:0/18:2)+NH_4_ | 0.136012991 |
| TG(16:0/16:0/18:1)+NH_4_ | 0.0215118 |
| TG(18:4/16:0/18:2)+H | 0.006794066 |
| TG(18:0/16:0/16:0)+NH_4_ | 0.004779449 |
| TG(16:0/18:2/18:3)+H | 0.013212064 |
| TG(16:0/18:2/18:3)+H | 0.008684219 |
| TG(15:0/18:3/18:3)+NH_4_ | 0.005557641 |
| TG(18:4/15:0/18:2)+NH_4_ | 0.003713274 |
| TG(16:0/18:1/18:3)+H | 0.01792111 |
| TG(15:0/18:2/18:3)+NH_4_ | 0.024584369 |
| TG(15:0/18:1/18:3)+NH_4_ | 0.055333955 |
| TG(18:4/15:0/18:2)+Na | 0.003787769 |
| TG(15:0/18:1/18:2)+NH_4_ | 0.012308344 |
| TG(16:0/17:1/18:2)+NH_4_ | 0.003389018 |
| TG(15:0/18:2/18:3)+Na | 0.019630558 |
| TG(16:0/17:0/18:2)+NH_4_ | 0.008850816 |
| TG(16:0/17:0/18:1)+NH_4_ | 0.002745281 |
| TG(18:4/16:0/18:3)+NH_4_ | 0.017448172 |
| TG(18:4/16:0/18:2)+NH_4_ | 0.118689643 |
| TG(16:1/18:2/18:3)+NH_4_ | 0.010545266 |
| TG(16:0/18:2/18:3)+NH_4_ | 0.00110153 |
| TG(16:1/18:2/18:2)+NH_4_ | 0.217462619 |
| TG(16:0/18:2/18:3)+NH_4_ | 0.063343156 |
| TG(16:1/18:2/18:3)+Na | 0.007835147 |
| TG(16:0/18:1/18:2)+NH_4_ | 0.212774378 |
| TG(16:0/18:2/18:3)+Na | 0.041975183 |
| TG(16:1/18:2/20:5)+H | 0.077596493 |
| TG(18:4/18:2/18:2)+H | 0.020230251 |
| TG(16:0/18:1/18:1)+NH_4_ | 0.065277212 |
| TG(18:3/18:2/18:2)+H | 0.030535729 |
| TG(18:3/18:2/18:2)+H | 0.010764464 |
| TG(16:1/18:1/20:5)+H | 0.004265457 |
| TG(16:1/18:1/18:2)+Na | 0.010988899 |
| TG(18:4/17:0/18:4)+NH_4_ | 0.010088337 |
| TG(18:0/16:0/18:1)+NH_4_ | 0.025195988 |
| TG(18:1/18:2/18:3)+H | 0.013537395 |
| TG(18:2/18:2/18:2)+H | 0.02609278 |
| TG(16:0/18:1/18:2)+Na | 0.096130652 |
| TG(18:3/17:1/18:3)+NH_4_ | 0.018093095 |
| TG(18:0/16:0/18:0)+NH_4_ | 0.00519486 |
| TG(18:3/17:1/18:2)+NH_4_ | 0.042380633 |
| TG(18:0/16:0/18:1)+Na | 0.010361542 |
| TG(18:2/17:1/18:2)+NH_4_ | 0.02910084 |
| TG(18:1/17:1/18:2)+NH_4_ | 0.039608459 |
| TG(17:0/18:2/18:2)+NH_4_ | 0.019135993 |
| TG(18:3/17:1/18:2)+Na | 0.033378201 |
| TG(17:0/18:1/18:2)+NH_4_ | 0.009310399 |
| TG(18:4/18:2/18:3)+NH_4_ | 0.083417278 |
| TG(18:3/18:3/18:3)+NH_4_ | 0.023616902 |
| TG(17:0/18:1/18:1)+NH_4_ | 0.005145598 |
| TG(18:1/17:1/20:5)+H | 0.024507764 |
| TG(18:3/18:2/18:3)+NH_4_ | 0.136581359 |
| TG(18:4/18:2/18:2)+NH_4_ | 0.25824376 |
| TG(19:1/16:0/18:2)+Na | 0.005753803 |
| TG(17:0/18:1/18:2)+Na | 0.005103965 |
| TG(18:3/18:2/18:2)+NH_4_ | 1.006319348 |
| TG(18:4/18:3/20:5)+H | 0.023212379 |
| TG(18:3/18:3/18:3)+Na | 0.006706332 |
| TG(16:0/18:2/19:0)+Na | 0.018032644 |
| TG(18:4/18:1/18:1)+NH_4_ | 0.039974636 |
| TG(18:3/18:2/18:3)+Na | 0.091174576 |
| TG(18:4/18:2/18:2)+Na | 0.103260856 |
| TG(18:1/18:1/18:2)+NH_4_ | 0.22473701 |
| TG(18:0/18:1/18:2)+NH_4_ | 0.0844931 |
| TG(18:1/18:2/18:2)+Na | 0.304313237 |
| TG(18:0/18:0/18:2)+NH_4_ | 0.02463831 |
| TG(18:1/18:1/18:2)+Na | 0.095198992 |
| TG(18:0/18:0/18:1)+NH_4_ | 0.005342118 |
| TG(18:0/18:1/18:2)+Na | 0.035713306 |
| TG(18:0/18:0/18:0)+NH_4_ | 0.002774465 |
| TG(19:1/18:2/18:3)+NH_4_ | 0.00958049 |
| TG(19:1/18:2/18:2)+NH_4_ | 0.012662363 |
| TG(19:0/18:2/18:2)+NH_4_ | 0.009767967 |
| TG(19:1/18:2/18:3)+Na | 0.00623913 |
| TG(15:0/18:2/22:1)+NH_4_ | 0.002785376 |
| TG(19:1/18:2/20:5)+H | 0.008336174 |
| TG(16:0/18:2/21:0)+NH_4_ | 0.002569515 |
| TG(19:0/18:2/18:2)+Na | 0.004292574 |
| TG(18:3/18:2/20:2)+NH_4_ | 0.032598786 |
| TG(56:7)+NH_4_ | 0.225625594 |
| TG(20:2/18:2/18:2)+NH_4_ | 0.499245446 |
| TG(20:2/18:2/18:2)+NH_4_ | 0.103509458 |
| TG(20:1/18:2/18:2)+NH_4_ | 0.155869726 |
| TG(20:1/18:2/18:2)+NH_4_ | 0.096081481 |
| TG(20:1/18:1/18:2)+NH_4_ | 0.027431872 |
| TG(57:3p)+NH_4_ | 0.002026895 |
| TG(20:2/18:2/20:5)+H | 0.051230075 |
| TG(20:0/18:1/18:2)+NH_4_ | 0.01248327 |
| TG(20:1/18:2/20:5)+H | 0.007288955 |
| TG(20:1/18:2/20:5)+H | 0.041511161 |
| TG(16:0/18:1/22:1)+NH_4_ | 0.008773944 |
| TG(20:0/18:2/20:5)+H | 0.018939343 |
| TG(16:0/18:1/22:0)+NH_4_ | 0.001352274 |
| TG(16:0/18:1/22:1)+Na | 0.002485367 |
| TG(18:3/18:2/21:0)+NH_4_ | 0.001154998 |
| TG(18:2/18:2/21:0)+NH_4_ | 0.007132007 |
| TG(16:0/18:2/23:1)+NH_4_ | 0.002377149 |
| TG(32:2e)+Na | 0.010138464 |
| TG(16:0/18:2/23:0)+NH_4_ | 0.005816411 |
| TG(18:2/18:2/21:0)+Na | 0.002760058 |
| TG(58:6)+NH_4_ | 0.017405048 |
| TG(22:1/18:2/18:2)+NH_4_ | 0.013982525 |
| TG(22:0/18:2/18:3)+NH_4_ | 0.005401383 |
| TG(22:0/18:2/18:2)+NH_4_ | 0.02227661 |
| TG(18:1/18:1/22:1)+NH_4_ | 0.004789161 |
| TG(16:0/18:2/24:0)+NH_4_ | 0.008034221 |
| TG(22:0/18:2/18:2)+Na | 0.008705443 |
| TG(12:0e/10:2/12:2)+H | 0.009990501 |
| TG(16:0/20:5/24:0)+H | 0.001863538 |
| TG(18:3/18:2/23:0)+NH_4_ | 0.004414531 |
| TG(18:2/18:2/23:0)+NH_4_ | 0.019563855 |
| TG(18:1/18:2/23:0)+NH_4_ | 0.00320824 |
| TG(18:2/18:2/23:0)+Na | 0.005550995 |
| TG(24:2/18:2/18:2)+NH_4_ | 0.001067005 |
| TG(24:0/18:2/18:3)+NH_4_ | 0.00646823 |
| TG(18:1/18:2/24:1)+NH_4_ | 0.023806688 |
| TG(18:1/18:2/24:0)+NH_4_ | 0.004858734 |
| TG(25:1/18:2/18:2)+NH_4_ | 0.001926731 |
| TG(25:0/18:2/18:2)+NH_4_ | 0.008649877 |
| TG(26:0/18:2/18:2)+NH_4_ | 0.002601541 |
| TG(16:0/8:0/10:4)+H | 0.002641063 |
| TG(6:0/12:2/16:0)+H | 0.007247326 |
| TG(16:0/8:0/10:2)+H | 0.012280732 |
| TG(6:0/12:2/16:0)+H | 0.004214254 |
| TG(6:0/12:2/16:1)+NH_4_ | 0.005448186 |
| TG(6:0/12:1/16:0)+NH_4_ | 0.016479266 |
| **Total TG** | 12.77131397 |
| WE(23:3)+NH_4_ | 0.00943028 |
| WE(40:5)-H | 0.008898069 |
| WE(21:1)+NH_4_ | 0.004705767 |
| WE(6:0/16:3)+NH_4_ | 0.012667125 |
| **Total WE** | 0.035701242 |

**Table S2**. Proteins found in *Panax notoginseng*-derived exosome-like nanoparticles and subcellular localization, as assessed by proteomic analysis.

| Protein | Protein namee | Subcellular localization |
| --- | --- | --- |
| A0A0D5ZDE5 | Glycosyltransferase | PlasmaMembrane;Cytoplasmic |
| A0A3S9H6G7 | Cytochrome P450 CYP736A225 | PlasmaMembrane;Mitochondrial;Chloroplast |
| Q0PVD2 | rRNA N-glycosidase | PlasmaMembrane |
| A0A3Q9BH58 | Cytochrome P450 CYP81BJ5 | Mitochondrial;Chloroplast |
| F4YF69 | Glycosyltransferase | Mitochondrial |
| A0A514EJD7 | SNF1-like protein kinase 3 | Cytoplasmic;Nuclear |
| A5Z0F9 | Calcium-dependent protein kinase 2 (Fragment) | Cytoplasmic;Mitochondrial |
| A0A0D5ZD66 | UGTPg33 | Cytoplasmic |
| A0A060IGT8 | Putative polyubiquitin | Cytoplasmic |
| A9UFP4 | Somatic embryogenesis protein kinase 3 (Fragment) | Mitochondrial |
| A0A0A7H8H0 | Glycosyltransferase | Cytoplasmic |
| A0A1P7Y0D9 | Pleiotropic drug resistance transporter (Fragment) | PlasmaMembrane |
| W8CQ86 | 50S ribosomal protein L16, chloroplastic | Mitochondrial |
| A0A5J6BVK7 | NADPH--cytochrome P450 reductase | ER |
| A0PGP7 | Specific abundant protein 3 | Extracellular |
| Q1ZZM9 | Phenylalanine ammonia-lyase 1 (Fragment) | Mitochondrial;Nuclear |
| A0A0D5ZCS9 | UGTPg17 | PlasmaMembrane;Cytoplasmic;Chloroplast |
| A0A514LVP4 | 30S ribosomal protein S2, chloroplastic | Mitochondrial |
| O82139 | Cycloartenol Synthase | Cytoplasmic;Lysosomal |
| A0A0D5ZDD9 | Glycosyltransferase | Cytoplasmic |
| F4YF80 | Cytochrome P450 | Cytoplasmic |
| A0A068JAV3 | Glycosyltransferase | Cytoplasmic;Chloroplast |
| A0A514LYL5 | ATP synthase subunit b, chloroplastic | Chloroplast |
| W8CPY8 | Photosystem I reaction center subunit VIII | Mitochondrial |
| W8CQB0 | Protein Ycf2 | Nuclear |
| W8CR46 | 50S ribosomal protein L22, chloroplastic | Mitochondrial;Nuclear |
| B0FPA2 | Terpene cyclase/mutase family member | Lysosomal |
| A0A0Y0AX26 | Eukaryotic translation initiation factor 3G1 (Fragment) | Cytoplasmic;Nuclear |
| W8CQ90 | 30S ribosomal protein S3, chloroplastic | Chloroplast |
| A0A3S8TLH9 | Gibberellin binding protein | PlasmaMembrane;Chloroplast |
| A0A0D5ZDD5 | Glycosyltransferase | PlasmaMembrane;Chloroplast |
| Q20BN1 | Cytochrome b6-f complex iron-sulfur subunit | Chloroplast |
| A0A0U2SDA5 | Chlorophyll a-b binding protein, chloroplastic | Chloroplast |
| A0A290GN01 | Superoxide dismutase (Fragment) | Mitochondrial |
| H2DH17 | Cytochrome P450 CYP749A22 | PlasmaMembrane |
| W8CR62 | 50S ribosomal protein L20, chloroplastic | Mitochondrial;Nuclear |
| A0A0A0YMU6 | Eukaryotic translation initiation factor (Fragment) | Nuclear |
| H2DH22 | Cytochrome P450 CYP73A100 | PlasmaMembrane |
| S5VGV8 | Terpene cyclase/mutase family member | Cytoplasmic |
| I7CT85 | Protopanaxadiol 6-hydroxylase | PlasmaMembrane |
| A0A385CIM2 | Glycosyltransferase | Cytoplasmic |
| Q1HGF2 | Dehydrin 6 (Fragment) | Nuclear |
| Q68RY2 | ATP-dependent Clp protease proteolytic subunit | Chloroplast |
| W8CQ94 | ATP synthase epsilon chain, chloroplastic | Chloroplast |
| W8CQX5 | 50S ribosomal protein L14, chloroplastic | Chloroplast |
| A0A096XF93 | NADH-ubiquinone oxidoreductase chain 1 | PlasmaMembrane |
| W8CQ66 | Cytochrome b559 subunit alpha | PlasmaMembrane |
| O47413 | NADH-dehydrogenase subunit 2 (Fragment) | PlasmaMembrane |
| Q8H9B4 | UDP-glucose:sterol 3-O-glucosyltransferase | Cytoplasmic |
| A0A0B4MQG7 | Acetyl-coenzyme A carboxylase carboxyl transferase subunit beta, chloroplastic | PlasmaMembrane |
| A0A140IEX7 | Ribosomal protein S8 (Fragment) | Mitochondrial;Chloroplast;Nuclear |
| A0A0K0PVL3 | UDP-glucosyltransferase 103 | PlasmaMembrane;Cytoplasmic |
| A0A0A7H7P4 | Glycosyltransferase | Cytoplasmic |
| A0A0R6ATQ1 | Pathogenesis-related protein | Cytoplasmic;Chloroplast |
| B6DMH0 | Cinnamyl alcohol dehydrogenase (Fragment) | Cytoplasmic;Mitochondrial |
| A0A0A6ZFR4 | UDP-glucosyltransferase 74AE2 | Cytoplasmic |
| F4YF78 | Cytochrome P450 | PlasmaMembrane |
| F4YF75 | Cytochrome P450 | Cytoplasmic;Mitochondrial |
| A0A0A0YMC4 | Eukaryotic translation initiation factor 5A | Nuclear |
| A0A0A0YQE8 | Actin (Fragment) | Cytoplasmic |
| D3W5F0 | Ribulose bisphosphate carboxylase large chain (Fragment) | Cytoplasmic;Chloroplast |
| I7BX75 | Glycosyltransferase | PlasmaMembrane |
| A0A096XF77 | Cytochrome b | PlasmaMembrane |
| A0A076JQL6 | Pathogenesis-related protein 10 | Cytoplasmic |
| H2DH19 | Cytochrome P450 CYP71D312 | Mitochondrial;Chloroplast |
| Q5I681 | Calcium-dependent/calmodulin-independent protein kinase (Fragment) | Cytoplasmic |
| A0A0U3US15 | 26S proteasome non-ATPase regulatory subunit 14-like protein (Fragment) | Mitochondrial;Nuclear |
| A0A514EJU0 | SNF1-like protein kinase 1 | Nuclear |
| F4YF65 | Dolichyl-diphosphooligosaccharide--protein glycosyltransferase subunit DAD1 | PlasmaMembrane |
| A0A514EJD9 | PYR1-like protein 4 | Cytoplasmic;Nuclear |
| W8CR06 | 30S ribosomal protein S7, chloroplastic | Mitochondrial;Nuclear |
| W8CR66 | 30S ribosomal protein S4, chloroplastic | Mitochondrial;Nuclear |
| Q20BM8 | Ribosomal_L6e_N domain-containing protein | Nuclear |
| A0A1D8MII2 | Transcription factor WRKY1 | Nuclear |
| Q8H9B5 | UDP-glucose:sterol 3-O-glucosyltransferase | Cytoplasmic |
| A0A068J4Z9 | UGT3 | Cytoplasmic;Chloroplast |
| Q002B6 | Calcium-dependent protein kinase 3 (Fragment) | Cytoplasmic |
| F4YF66 | Glycosyltransferase | PlasmaMembrane;Cytoplasmic |
| A0A166JH20 | Peptidyl-prolyl cis-trans isomerase | Cytoplasmic |
| A0A060L489 | NADPH--cytochrome P450 reductase | ER |
| A0A076JP87 | Aluminum-induced protein | Chloroplast |
| B3GLJ1 | S-adenosylmethionine synthase | Cytoplasmic |
| A0A060IMP7 | Putative GTP-binding protein sar1 (Fragment) | Extracellular |
| A0A096XF70 | NADH dehydrogenase subunit 7 | Mitochondrial |
| A0A076V5N5 | Phosphomevalonate kinase | Extracellular |
| W8FMB6 | Pathogenesis-related protein 4 | Extracellular |
| A0A0D5ZD63 | Glycosyltransferase | Extracellular;Cytoplasmic |
| A0A3Q9BGT6 | Cytochrome P450 CYP81B102 | PlasmaMembrane |
| D1MEN6 | Calcium-dependent protein kinase 1 | Cytoplasmic;Nuclear |
| A0A0B4MQQ6 | Cytochrome f | Chloroplast |
| B2MVQ2 | Glutathione S-transferase | Cytoplasmic |
| A0A0G2RE83 | Cytochrome b6 | PlasmaMembrane |
| Q8RVT5 | Acyl-CoA-binding protein | Cytoplasmic;Nuclear |
| A0A060ID14 | Cyclophilin (Fragment) | Mitochondrial |
| W8CR01 | Photosystem I P700 chlorophyll a apoprotein A2 | PlasmaMembrane |
| A0A0Y0C5Z0 | Eukaryotic translation initiation factor 3 subunit B (Fragment) | Cytoplasmic |
| Q6JSK3 | Betaine aldehyde dehydrogenase | Cytoplasmic |
| A0A3S9H6N0 | Cytochrome P450 CYP82D176 | PlasmaMembrane |
| F4YF79 | Cytochrome P450 | Cytoplasmic;Mitochondrial |
| Q6XY45 | Granule-bound starch synthase I (Fragment) | Cytoplasmic |
| F4YF72 | Cytochrome P450 | Chloroplast |
| A0A096XF91 | ATPase subunit 8 | PlasmaMembrane |
| D0UU71 | Glycolate oxidase | Cytoplasmic;Mitochondrial |
| A0A0D5ZDC4 | Glycosyltransferase | Cytoplasmic |
| A0A0D5ZD60 | Glycosyltransferase | Chloroplast |
| A0A411DVG2 | Dirigent protein | Extracellular |
| F4YF67 | Glycosyltransferase | Cytoplasmic |
| O22668 | Superoxide dismutase [Cu-Zn] | Cytoplasmic |
| A0A060IMR4 | Translationally controlled tumor protein | Cytoplasmic |
| W8CR36 | Photosystem I P700 chlorophyll a apoprotein A1 | PlasmaMembrane |
| W8CR23 | Photosystem II protein D1 | PlasmaMembrane |
| D2K762 | Squalene synthase 3 | PlasmaMembrane |
| Q9MAV8 | 60S ribosomal protein L27 | Cytoplasmic;Mitochondrial |
| A0A060IGE4 | 30S ribosomal protein S20 (Fragment) | Nuclear |
| A0A076VAK4 | Isopentenyl diphosphate isomerase 2 | Extracellular;Chloroplast |
| Q6SSE2 | Photosystem II D2 protein | PlasmaMembrane |
| A0A096XF79 | NADH dehydrogenase subunit 9 | Nuclear |
| A0A068ESC4 | Signal peptidase complex subunit 3B (Fragment) | Cytoplasmic;Mitochondrial |
| A9QXE9 | Glutaredoxin | Extracellular;Cytoplasmic;Chloroplast |
| A0A0A6ZFY4 | UDP-glucosyltransferase 29 | Cytoplasmic;Chloroplast |
| I6UXL1 | Mevalonate kinase | PlasmaMembrane;Cytoplasmic;Chloroplast |
| A0A096XF73 | ATPase subunit 4 | Nuclear |
| C1K2M3 | Class 1 chitinase | Extracellular |
| A0A0A0YLE9 | Tubulin alpha chain | Cytoplasmic |
| F4YF74 | Cytochrome P450 | PlasmaMembrane |
| A9QMA1 | Thioredoxin h-like protein (Fragment) | Cytoplasmic |
| A0A076V637 | Diphosphomevalonate decarboxylase | PlasmaMembrane |
| A0A0D5ZDJ5 | Glycosyltransferase | Cytoplasmic |
| B8YDG5 | Short-chain alcohol dehydrogenase | Cytoplasmic |
| E9M220 | PR10-3 (Fragment) | Cytoplasmic |
| A0A060ILM1 | Ubiquinol-cytochrome C reductase iron-sulfur subunit | Chloroplast |
| A0A411DVA3 | Beta-1,3-glucanase | Vacuole |
| A0A0Y0AXD6 | Actin (Fragment) | Cytoplasmic |
| Q68RZ7 | Acetyl-coenzyme A carboxylase carboxyl transferase subunit beta, chloroplastic | PlasmaMembrane |
| A0A3Q9BGP9 | Cytochrome P450 CYP80F5 | Cytoplasmic;Mitochondrial |
| A0A514LYJ1 | Protein TIC 214 | Nuclear |
| A0A0H3V0I8 | Protein TIC 214 | Nuclear |
| F4YF70 | Cytochrome P450 | Chloroplast |
| A0A220QKD0 | Ascorbate peroxidase | Cytoplasmic |
| Q75W19 | Cytochrome P450 | Cytoplasmic;Mitochondrial |
| A0A068J5R0 | Glycosyltransferase | Chloroplast |
| H2DH20 | Cytochrome P450 CYP71D313 | Cytoplasmic |
| Q20BM6 | Ribosomal protein L31 | Cytoplasmic;Nuclear |
| A0A4D6DD35 | Class 1 chitinase | Extracellular |
| A0A060IGR7 | Elongation factor 1-alpha (Fragment) | Cytoplasmic |
| C1KGC1 | Calmodulin | Cytoplasmic |
| H2DH21 | Cytochrome P450 CYP72A219 | PlasmaMembrane |
| P83618 | Ribonuclease-like storage protein | Extracellular |
| F4YF71 | Cytochrome P450 | Mitochondrial |
| Q68RU2 | 50S ribosomal protein L2, chloroplastic | Mitochondrial;Nuclear |
| A0A221HZJ8 | Pathogensis-related protein 10 | Cytoplasmic |
| Q20BN2 | ADP-ribosylation factor-like protein | Cytoplasmic;Mitochondrial;Chloroplast |
| A0A060IGE0 | Tubulin beta chain (Fragment) | Cytoplasmic |
| S5RMQ6 | Sesquiterpene synthase | Cytoplasmic |
| U5NNF1 | Glutathione peroxidase | Extracellular |
| A0A513WY80 | PR10-3 | Chloroplast |
| U5KP94 | Pleiotropic drug resistance transporter 3 | PlasmaMembrane |
| P80889 | Ribonuclease 1 | Cytoplasmic |
| A0A0Y0AX85 | Elongation factor 1-beta | Cytoplasmic |
| A0A0Y0AU34 | Elongation factor 1-gamma (Fragment) | Cytoplasmic;Mitochondrial;Chloroplast |
| Q9MAW6 | 60S ribosomal protein L27a | Cytoplasmic;Nuclear |
| W8CQA1 | Photosystem II CP43 reaction center protein | PlasmaMembrane |
| A0A220QKC7 | Ascorbate peroxidase | Cytoplasmic |
| A0A076VAK8 | Acetyl-CoA C-acetyltransferase | Chloroplast |
| W8CR09 | ATP synthase subunit alpha, chloroplastic | Chloroplast |
| A0A3S9H6I3 | Cytochrome P450 CYP94Q2 | PlasmaMembrane |
| A0A060IMQ8 | Eukaryotic translation initiation factor 5A | Cytoplasmic |
| D7RWP9 | Peroxiredoxin | Cytoplasmic |
| Q1HGF5 | Dehydrin 3 | Nuclear |
| U5NNJ1 | Glutathione peroxidase | Mitochondrial;Chloroplast |
| A0A0B4MQQ0 | Ribulose bisphosphate carboxylase large chain | Chloroplast |
| S5W638 | Farnesyl diphosphate synthase | Cytoplasmic |
| Q307T0 | Tonoplast intrinsic protein | PlasmaMembrane |
| W8CQM8 | Photosystem II CP47 reaction center protein | PlasmaMembrane |
| A0A0K0PVM5 | UDP-glycosyltransferase 101 | PlasmaMembrane;Cytoplasmic |
| A0A060ID18 | ADP-ribosylation factor (Fragment) | Cytoplasmic;Mitochondrial;Nuclear |
| O22669 | Chlorophyll a-b binding protein, chloroplastic | Chloroplast |
| A9QMA3 | 60S ribosomal protein L17-like protein (Fragment) | Mitochondrial |
| D0VNY8 | Pathogensis-related protein 10 | Cytoplasmic |
| A0A096VPN4 | Pleiotropic drug resistance transporter 2 | PlasmaMembrane |
| W8CQX6 | ATP synthase subunit beta, chloroplastic | Chloroplast |
| Q3LRW8 | Glyceraldehyde-3-phosphate dehydrogenase (Fragment) | Cytoplasmic |
| A9Z0Q0 | Catalase | Peroxisomal |
| A0A068J5Q3 | Glycosyltransferase | Cytoplasmic |
| Q1HGF4 | Dehydrin 4 | Nuclear |
| A0A0A0YSN6 | Tubulin beta chain | Cytoplasmic |
| A0A060ID32 | Tubulin alpha chain | Cytoplasmic |
| Q3LFQ4 | Ribulose bisphosphate carboxylase small chain | Chloroplast |
| A0A0A0YMC6 | Ubiquitin (Fragment) | Nuclear |
| A0A059T4E0 | Cytochrome P450 | PlasmaMembrane |
| A0A0A0YLE5 | Peptidyl-prolyl cis-trans isomerase | Cytoplasmic |
| A0A060ILK6 | Putative V-type proton ATPase subunit B 1 (Fragment) | Cytoplasmic;Nuclear |
| D3JX88 | Glutamate decarboxylase | Cytoplasmic |
| B5THI3 | Major latex-like protein | Cytoplasmic |
| Q6VAL5 | Glyceraldehyde-3-phosphate dehydrogenase | Cytoplasmic |
| A0A060IGE8 | 60S ribosomal protein L13a (Fragment) | Mitochondrial |
| Q9MAV9 | Cytoplasmic ribosomal protein S13 | Mitochondrial |
| A0A096VPM7 | Pleiotropic drug resistance transporter 1 | PlasmaMembrane |
| O47412 | ATP synthase subunit alpha | Mitochondrial |
| A0A0A0YSN1 | Elongation factor 1-alpha | Cytoplasmic |
| A0A060IMN8 | Actin 1 | Cytoplasmic |
| Q401B8 | 40S ribosomal protein S4 | Cytoplasmic;Mitochondrial |
| Q20BN0 | BBE domain-containing protein | PlasmaMembrane |
| W8CR60 | Ribulose bisphosphate carboxylase large chain | Chloroplast |
| A0A0A0YQF5 | Glyceraldehyde-3-phosphate dehydrogenase | Cytoplasmic |
| X2D3J9 | RNase-like major storage protein | Extracellular |

**Table S3.** miRNAs from PDN.

| **Name** | **Sequence** | **Readcount** |
| --- | --- | --- |
| novel_2 | UUGGGACAUCUGAAGGAACGGG | 1 |
| novel_4 | GGUGAUGCCGUAAUUCCAGAA | 1 |
| ath-miR157a-5p | UUGACAGAAGAUAGAGAGCAC | 1 |
| ath-miR8175 | GAUCCCCGGCAACGGCGCCA | 1 |
| csi-miR156d-5p | UGACAGAAGAUAGAGAGCGC | 1 |
| hbr-miR156 | UUGACAGAAGAUAGAGAGC | 1 |
| hci-miR164a | UGGAGAAGCAGGGCACGUGAA | 1 |
| mdm-miR391 | UACGCAGGAGAGAUGACGCCG | 1 |
| osa-miR164e | UGGAGAAGCAGGGCACGUGAG | 1 |
| pgi-miR482a | UCUUGCCAAUUCCUCCCAUUCC | 1 |
| ath-miR159b-3p | UUUGGAUUGAAGGGAGCUCUU | 2 |
| bdi-miR159a-3p | CUUGGAUUGAAGGGAGCUCU | 2 |
| fve-miR159b | AUUGGAUUGAAGGGAGCUCUC | 2 |
| gma-miR4995 | AGGCAGUGGCUUGGUUAAGGG | 2 |
| lus-miR159b | UUUGGAUUGAAGGGAGCUCUC | 2 |
| osa-miR159a.1 | UUUGGAUUGAAGGGAGCUCUG | 2 |
| rgl-miR5141 | AGACCCGACGCGACUGACAGAUAA | 3 |
| novel_1 | UUUCAUGCCUAAACCAAGGGA | 3 |
| novel_3 | UUGACCAAUACAGUGCUGACACGC | 3 |
| zma-miR164h-5p | UGGAGAAGCAGGGCACGUGUG | 3 |
| pgi-miR6136a.2 | ACGGGUGAGUAAGAUAAGGGGUAU | 6 |
| aqc-miR159 | UUUGGACUGAAGGGAGCUCUA | 7 |
| ath-miR159a | UUUGGAUUGAAGGGAGCUCUA | 9 |
| osa-miR159f | CUUGGAUUGAAGGGAGCUCUA | 9 |
| sly-miR403-5p | CGUUUGUGCGUGAAUCUAACA | 9 |
| csi-miR403b-5p | AGUUUGUGCGUGAAUCUAACC | 10 |
| gma-miR403a | UUAGAUUCACGCACAAACUUG | 10 |
| ath-miR164a | UGGAGAAGCAGGGCACGUGCA | 34 |
| ath-miR164c-5p | UGGAGAAGCAGGGCACGUGCG | 34 |
| gma-miR164b | UGGAGAAGCAGGGCACGUGC | 34 |
| osa-miR164d | UGGAGAAGCAGGGCACGUGCU | 34 |
| ath-miR403-3p | UUAGAUUCACGCACAAACUCG | 35 |
| pgi-miR2118 | UUUCCUAUUCCACCCAUCCCAU | 43 |
| ath-miR156a-5p | UGACAGAAGAGAGUGAGCAC | 50 |
| cca-miR156b | UGACAGAAGAGAGUGAGCAUA | 50 |
| fve-miR156h | UGACAGAAGAGAGUGAGCUC | 50 |
| stu-miR156f-5p | CUGACAGAAGAGAGUGAGCA | 50 |
| ath-miR319a | UUGGACUGAAGGGAGCUCCCU | 185 |
| ath-miR319c | UUGGACUGAAGGGAGCUCCUU | 185 |
| gma-miR319c | UUGGACUGAAGGGAGCUCCU | 185 |
| gma-miR319q | UGGACUGAAGGGAGCUCCUUC | 185 |
| mtr-miR319a-3p | UUGGACUGAAGGGAGCUCCC | 185 |
| ppt-miR319a | CUUGGACUGAAGGGAGCUCC | 187 |
| pta-miR319 | UUGGACUGAAGGGAGCUCCC | 187 |

Full Version of blots


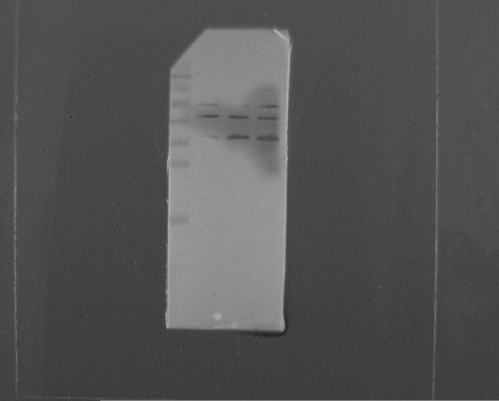


actin

**
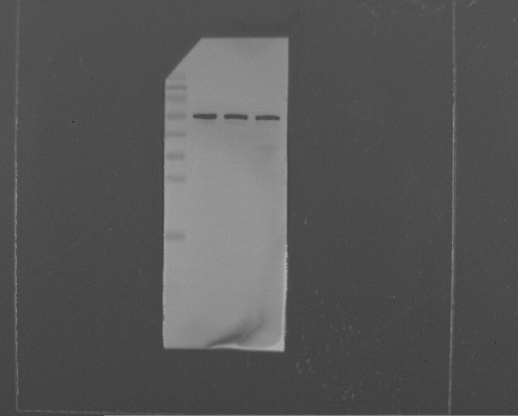
**

Akt


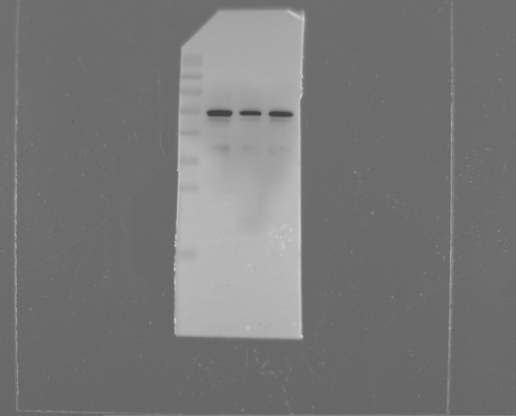


p-Akt


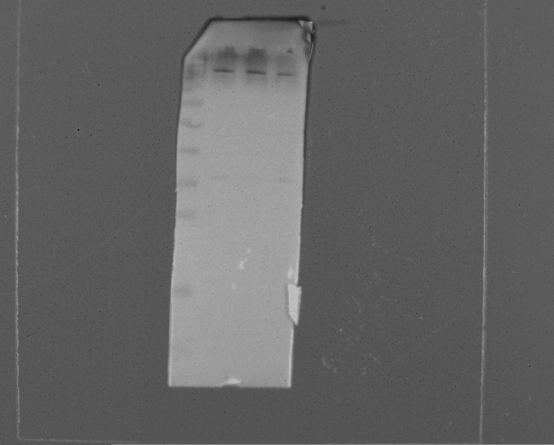


pI3k


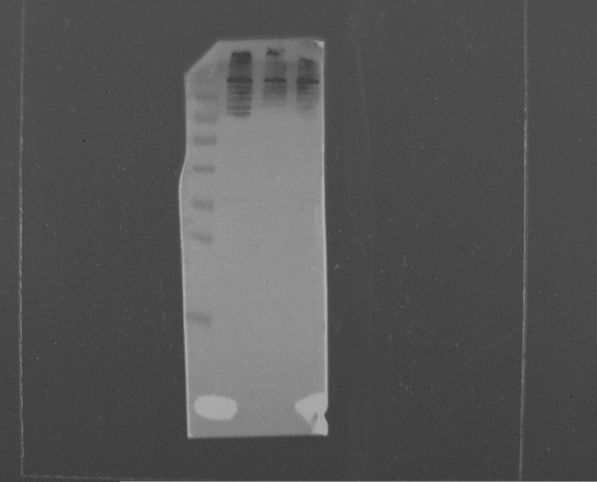


p-pI3k
